# The rs1001179 SNP and CpG methylation regulate catalase expression in chronic lymphocytic leukemia

**DOI:** 10.1007/s00018-022-04540-7

**Published:** 2022-09-16

**Authors:** Marilisa Galasso, Elisa Dalla Pozza, Roberto Chignola, Simona Gambino, Chiara Cavallini, Francesca Maria Quaglia, Ornella Lovato, Ilaria Dando, Giorgio Malpeli, Mauro Krampera, Massimo Donadelli, Maria G. Romanelli, Maria T. Scupoli

**Affiliations:** 1 Biology and Genetics Section, Department of Neurosciences, Biomedicine and Movement Sciences, University of Verona, Strada Le Grazie 8, 37134 Verona, Italy; 2 Section of Hematology, Department of Medicine, University of Verona, Policlinico G.B. Rossi, P. L.A. Scuro 10, 37134 Verona, Italy; 3 Department of Biotechnology, University of Verona, Strada Le Grazie 15, 37134 Verona, Italy; 4 Research Center LURM, University of Verona, Policlinico G.B. Rossi, P. L.A. Scuro 10, 37134 Verona, Italy; 5 Department of Surgery, Dentistry, Pediatrics, and Gynecology, University of Verona, Policlinico G.B. Rossi, P. L.A. Scuro 10, 37134 Verona, Italy

**Keywords:** Chronic lymphocytic leukemia, Catalase, Single nucleotide polymorphism, DNA methylation

## Abstract

Chronic lymphocytic leukemia (CLL) is an incurable disease characterized by an extremely variable clinical course. We have recently shown that high catalase (*CAT*) expression identifies patients with an aggressive clinical course. Elucidating mechanisms regulating *CAT* expression in CLL is preeminent to understand disease mechanisms and develop strategies for improving its clinical management. In this study, we investigated the role of the *CAT* promoter rs1001179 single nucleotide polymorphism (SNP) and of the CpG Island II methylation encompassing this SNP in the regulation of *CAT* expression in CLL. Leukemic cells harboring the rs1001179 SNP T allele exhibited a significantly higher *CAT* expression compared with cells bearing the CC genotype. *CAT* promoter harboring the T -but not C- allele was accessible to ETS-1 and GR-β transcription factors. Moreover, CLL cells exhibited lower methylation levels than normal B cells, in line with the higher CAT mRNA and protein expressed by CLL in comparison with normal B cells. Methylation levels at specific CpG sites negatively correlated with *CAT* levels in CLL cells. Inhibition of methyltransferase activity induced a significant increase in CAT levels, thus functionally validating the role of CpG methylation in regulating *CAT* expression in CLL. Finally, the CT/TT genotypes were associated with lower methylation and higher *CAT* levels, suggesting that the rs1001179 T allele and CpG methylation may interact in regulating *CAT* expression in CLL. This study identifies genetic and epigenetic mechanisms underlying differential expression of *CAT*, which could be of crucial relevance for the development of therapies targeting redox regulatory pathways in CLL.

**Graphical abstract:**

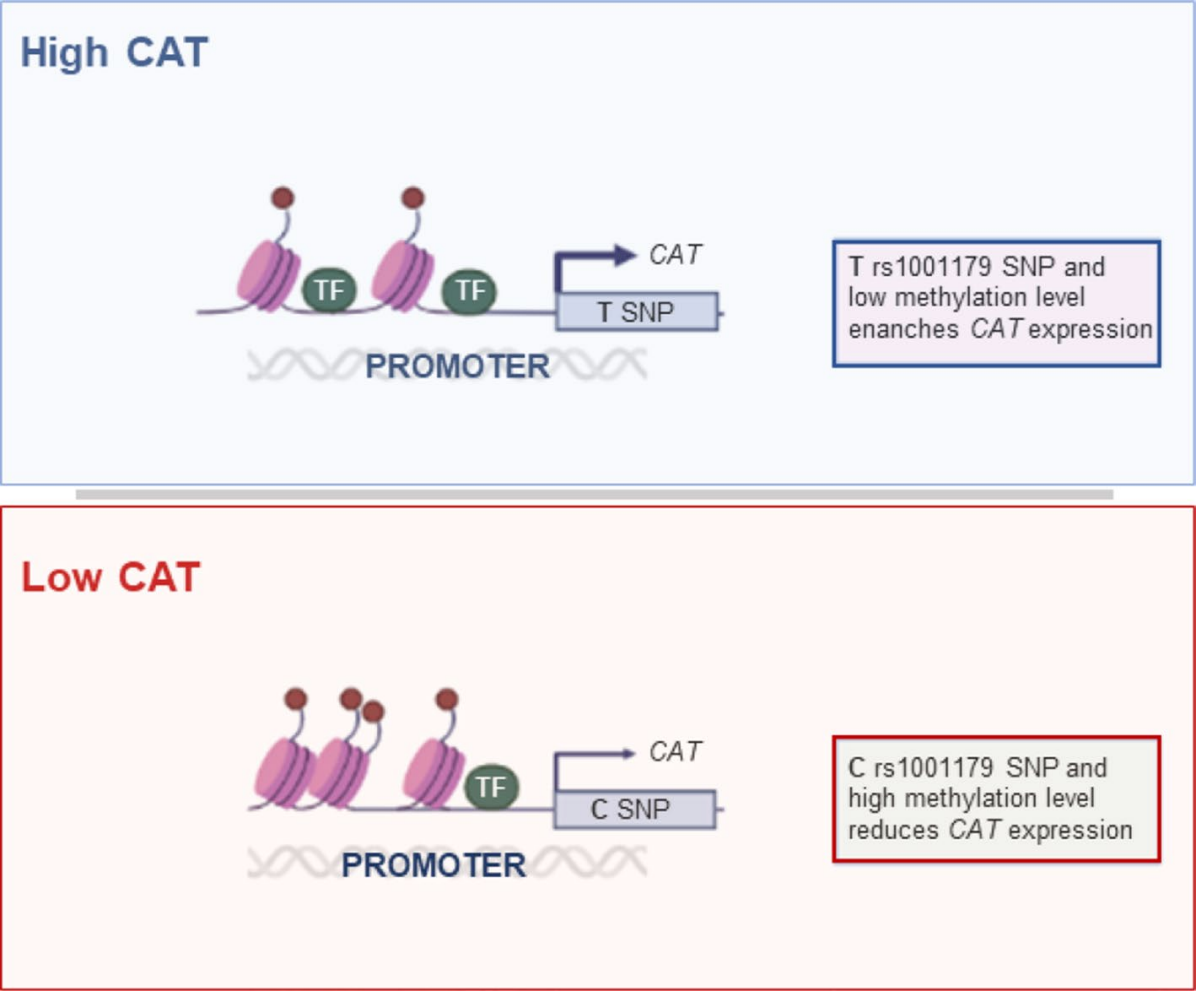

**Supplementary Information:**

The online version contains supplementary material available at 10.1007/s00018-022-04540-7.

## Introduction

Chronic lymphocytic leukemia (CLL), the most prevalent form of leukemia in Western countries, is an incurable disease exhibiting an extremely variable clinical course and response to treatment [1]. The disease is characterized by an accumulation of monoclonal, mature, CD5 + B cells in the peripheral blood, bone marrow, and secondary lymphoid organs [1]. In the last decades, advances in understanding the biological heterogeneity of the disease have led to the identification of proteins in signaling pathways involved in leukemia homing, survival, and proliferation [2]. Some of these proteins have been associated with a more aggressive form of the disease and are targets for novel therapeutic intervention [3]. However, there remains substantial intragroup clinical heterogeneity in otherwise molecularly homogeneous CLL subgroups [4]. Moreover, responses to novel therapies are heterogeneous across patients and resistance or toxicity associated with their long-term exposure are common phenomena [1].

Along with the understanding of molecular heterogeneity of the disease, growing interest is emerging in redox metabolism in CLL. Alterations of redox homeostasis have been often observed in cancer [5, 6]. Increased reactive oxygen species (ROS) levels have been detected in various cancers, where they activate protumorigenic signals; enhance cell survival, proliferation, and chemoresistance; and cause DNA damage and genetic instability [7, 8]. However, escalated levels of ROS can also promote ferroptosis and antitumorigenic signals, resulting in an increase of oxidative stress and induction of cancer cell death [7, 9]. In CLL, leukemic cells accumulate higher levels of ROS than normal B cells [10]. However, ROS levels are extremely variable across samples of patients and higher ROS levels are associated with favorable prognostic features and a slower disease progression [11]. Augmented levels of ROS also confer increased sensitivity to anticancer agents, inducing apoptosis in leukemia cells [12]. Thus, escalated levels of ROS seem to account for lesser aggressive behavior of CLL cells. Although the underlying mechanisms of altered ROS in cancer patients often remain elusive, alterations of the multifaced antioxidant enzyme system controlling ROS homeostasis have been observed in several cancers [13, 14]. Specifically, the crucial antioxidant enzyme catalase (CAT), which decomposes H_2_O_2_ to O_2_ and H_2_O, is often altered in cancer cells [15]. CAT can protect cells from tumor initiation and progression, due to its role in preventing the accumulation of dangerous levels of oxidants. In line with this, some studies have reported downregulation of CAT expression in some cancers [13, 14, 16]. However, CAT expression is highly expressed in other cancer cells, which require high antioxidant detoxifying systems and upregulation of CAT for tumor progression and metastasis to compensate for high ROS production and to prevent ROS-mediated cell death processes [10, 17, 18]. Consistently, we have recently documented that high *CAT* mRNA expression identifies an aggressive clinical course whereas low *CAT* levels are associated with an indolent disease in CLL [19]. This dichotomous expression of *CAT* in CLL subsets with divergent clinical behaviors highlights the importance to decipher the molecular mechanisms regulating *CAT* expression in leukemia cells.

Regulation of CAT expression in cancer is known to be multifactorial, including genetic and epigenetic changes, transcriptional and posttranscriptional regulations as well as posttranslational modifications [15]. However, the molecular mechanisms involved remain still poorly characterized in cancers. The human *CAT* gene core promoter is located in the first 200 bp region from the major *CAT* transcription start site [20]. The promoter, enriched in GC bases, contains multiple transcription start sites and both GGGCGG and CCAAT boxes, but lacks a TATA box and classical initiator element sequences [20, 21]. The human *CAT* gene is characterized by the presence of several single nucleotide polymorphisms (SNPs) in the promoter, 5′ and 3′-untranslated regions, exons and introns [15]. However, only the rs1001179 SNP in the *CAT* promoter, which consists of C > T substitution at 34,438,684 positions on chromosome 11 (GRCh38; − 330 position from ATG), alters CAT expression as well as blood CAT levels [22, 23, 24]. The human *CAT* gene also contains several CpG islands, among which the largest is the second one located between the promoter and the first exon [25]. Some evidence indicates that epigenetic changes, such as DNA methylation, contribute to the regulation of CAT expression in several biological contexts [26, 27].

In this study, we investigated mechanisms regulating *CAT* expression in leukemic cells of CLL patients. We identified the rs1001179 SNP and DNA methylation status as mechanisms involved in regulating *CAT* expression in CLL that could underlie differential *CAT* expression in subsets of patients.

## Materials and methods

### Cell samples

Peripheral blood mononuclear cells (PBMCs) from 75 untreated CLL patients and 55 age-matched healthy donors (HDs) were collected and cryopreserved at the Hematology Unit, Azienda Ospedaliera Universitaria Integrata in Verona (Italy) under a protocol approved by the local Ethics Committee. In accordance with the Declaration of Helsinki, all patients provided written informed consent for the collection and use of their blood samples for research purposes. The sample workflow is shown in Supplementary Fig. 1. Clinical annotations at diagnosis are summarized in Table S1. PBMCs were isolated and prepared as indicated in Online Supplementary Methods. MEC1 cell line (German Collection of Microorganisms and Cell Cultures—DSMZ, DE, EU) was maintained in IMDM; primary CLL cells and the mouse bone marrow-derived stromal cell line (M210B4, kindly provided by Dr Connie J Eaves, Terry Fox Laboratories, BC, CA) were maintained in RPMI 1640 (Thermo Fisher Scientific MA, USA). The culture media were supplemented with 10% heat-inactivated fetal bovine serum, 2 mM L-glutamine and 2 mM penicillin/streptomycin, at 37 °C in 5% CO_2_.

### Quantitative polymerase chain reaction

Quantitative polymerase chain reaction (qPCR) for *CAT, DNMTs* and *TETs* mRNA quantification was assessed using PowerUp SYBR Green Master Mix (Applied Biosystems, CA, USA). Samples were run in triplicate on the Real-Time Quant Studio 3 (Thermo Fisher Scientific), as detailed in Online Supplementary Methods.

### Flow cytometry

Protein expression levels of CAT were assessed using monoclonal antibodies (Table S2) and flow cytometry, as described in Online Supplementary Methods.

### DNA extraction and Genotyping

Genomic DNA extraction from CLL and HD PBMCs was performed using the salting-out method. Genotyping was assessed as previously described by Zarei et al. [28].

### Chromatin immunoprecipitation

Chromatin immunoprecipitation (ChIP) was performed according to the EpiQuik™ Chromatin Immunoprecipitation Kit (Epigentek, NY, USA), as previously described [29]. Briefly, cells were cross-linked with 1% formaldehyde. The cross-linked lysate was sonicated 10 times for 15 s interspersed by 30 s of rest on ice between each pulse to obtain average DNA fragment sizes ranging from 200 to 1000 bp. The sheared DNA was immunoprecipitated with the kit-provided Non-Immune IgG negative control, 4 µg of anti-ETS-1 (Santa Cruz, CA, USA), anti-GRβ (Abcam, CB, GB) and anti-STAT4 (Genetex International, CA, USA). The immunoprecipitated DNA quantification was performed amplifying the region of interest (from − 371 to − 255, human CAT promoter region location from ATG) using qPCR. The primers used were: CAT Chip F, 5′-AGGATGCTGATAACCGGGAG-3′; CAT Chip R, 5′-AGGGTGCGGAAAGGAAGG-3′. The thermal cycle reaction was performed as follows: 95 °C for 10 min followed by 40 cycles at 95 °C for 15 s and 60 °C for 1 min. The average cycle threshold of each triplicate was normalized to the input (un-immunoprecipitated DNA). Data are expressed as a percentage of input DNA that represents the enrichment of TFs on the specific region of CAT promoter surrounding rs1001179 SNP.

### Pyrosequencing

Quantification of methylation levels of eight CpG sites in the *CAT* promoter region (GRCh38 ( +)—Chr11: 34,438,657–34,438,708) was determined by pyrosequencing of bisulfite-converted DNA. Sample bisulfite treatment, PCR amplification, pyrosequencing, and quantification of methylation levels were performed by EpigenDx (MA, USA).

### Inhibition of DNA methyltransferase in CLL cells

MEC1 cells were seeded at 0.5 × 10^6^ cells/ml in culture media. HD B cells and primary CLL cells were added at a concentration of 1 × 10^6^ cells/ml to pre-seeded and sublethally irradiated M210B4 cells to support primary-cell survival. Cells were treated for 96 h in a medium containing DMSO vehicle or 2 µM DNA methyltransferase inhibitor 5-aza-2′-deoxycytidine (DAC; Sigma Aldrich, MO, USA). After treatment, *CAT* mRNA levels were assessed by qPCR, as previously described.

### Software and Statistical analysis

Hardy–Weinberg equilibrium was validated by *χ*^2^ test. Fisher’s exact test, unpaired Student’s t-test, Mann–Whitney, Wilcoxon matched-pairs signed rank test, and log-rank (Mantel-Cox) test were used where indicated. Time-to-first-treatment (TTFT) was calculated as previously described [19]. Correlation analysis was performed calculating the Spearman correlation coefficient (Spearman *r*). A *P* value < 0.05 was considered statistically significant. Graphing and statistical analyses were performed using GraphPad Prism software (v. 7.03, GraphPad Software Inc., CA, USA). Linear models were developed using the open-source platform for statistical computing R (version 3.6.0). *In-silico* analysis and mathematical models have been detailed in Online Supplementary Methods.

## Results

### Higher levels of catalase are associated with a faster leukemia progression

**Fig. 1:**
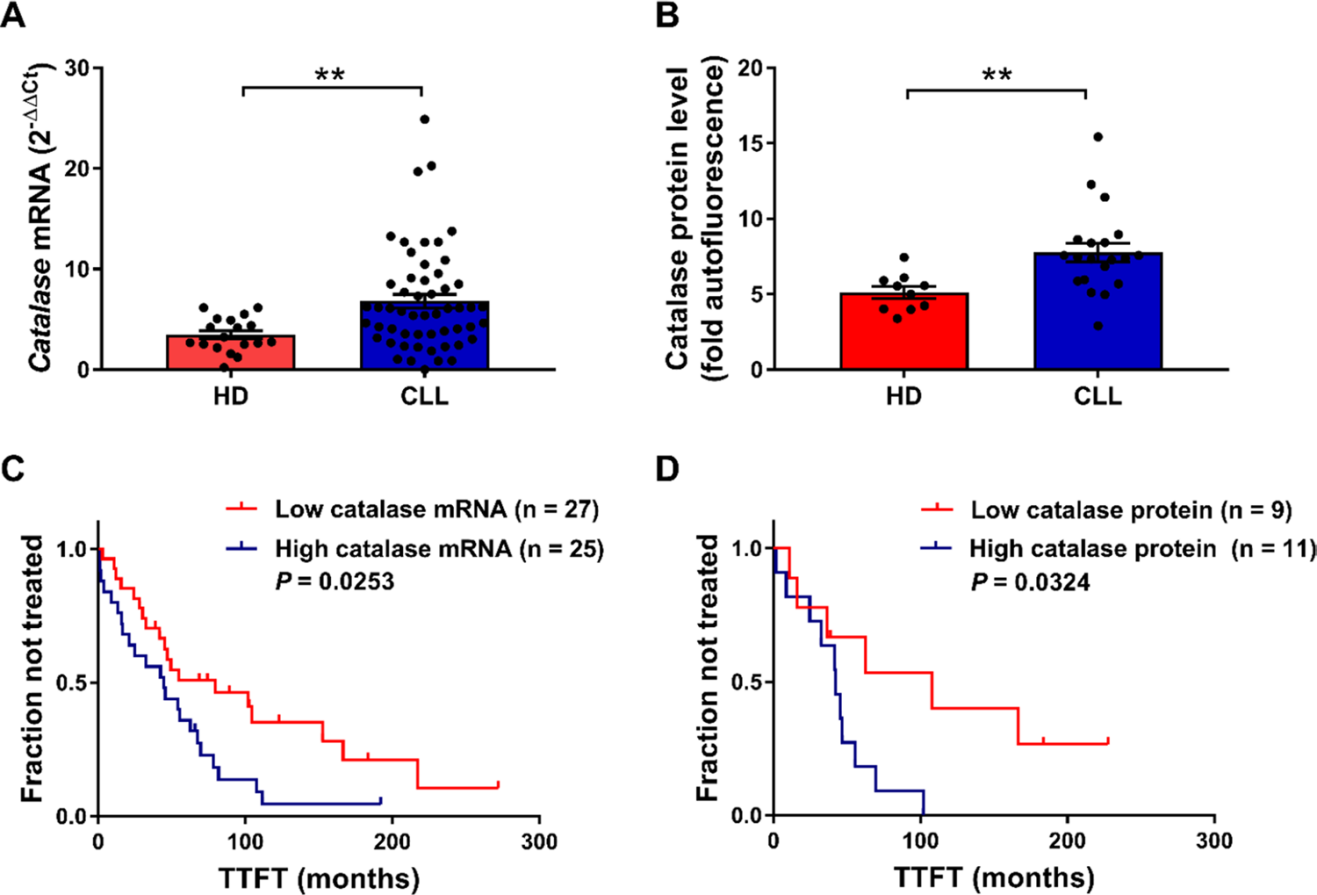
CAT expression level and its association with TTFT in CLL. **A** *CAT* mRNA levels in B cells purified from HD (*n* = 18) compared with B cells purified from CLL samples (*n* = 54). Data are expressed as relative quantification using comparative Ct method (2^–ΔΔCt^) and normalized to the expression value of the human embryonic kidney 293 cell line (HEK293) set as 1. **B** CAT protein expression in HD (*n* = 10) compared with CLL B-cell samples (*n* = 20). Data are expressed as fold autofluorescence calculated as median fluorescence intensity (MFI) divided by fluorescence-minus-one (FMO). Data are reported as mean ± SEM. Comparisons were performed with Mann Whitney test. **P < 0.01. **C** Kaplan–Meier curves of TTFT for subgroups of CLL patients distinguished by low (*n* = 27) and high (*n* = 25) *CAT* mRNA levels. **D** Kaplan–Meier curves of TTFT for subgroups of CLL patients distinguished by low (*n* = 9) and high (*n* = 11) CAT protein levels. High and low CAT expression values for both mRNA and protein were referred to the median expression values. Difference between the two curves was calculated with log-rank test. *TTFT* time to first treatment, *HD* healthy donor, *CLL* chronic lymphocytic leukemia

We have recently identified low *CAT* expression as a major antioxidant element that identifies an indolent clinical behavior in CLL whereas high *CAT* expression is associated with a more aggressive disease course [19]. To validate the prognostic significance of *CAT* expression in the patients’ sample set analyzed in this study, first we characterized CAT mRNA and protein expression in B cells isolated from CLL patients and HDs. Although the levels of *CAT* mRNA were highly heterogeneous among CLL samples (coefficients of variation: CV = 74.72% for CLL versus CV = 49.37% for HD B cells), CLL cells expressed higher average *CAT* mRNA levels compared with HD B cells (Fig. 1A), thus confirming previous data [10]. CLL cells also exhibited an overall higher and more heterogeneous CAT protein level than HD B cells (CV = 35.75 for CLL versus CV = 23.99% for HDs) (Fig. 1B). Association between CAT mRNA and protein levels in CLL B cells is shown in Supplementary Fig. 2.

Then, we aimed at validating the association of catalase expression and disease behavior in the analyzed patients’ sample set. As shown in Fig. 1C, Kaplan–Meier curves showed that high levels of *CAT* mRNA were significantly associated with a faster disease, indicated as a shorter time to first treatment (TTFT). Moreover, we confirmed the association between CAT expression and disease progression also at a protein level (Fig. 1D). In contrast, we detected no association between CAT expression, either at the mRNA and protein levels, and the *IGHV*-gene mutational status, the most reliable biological prognostic factor in CLL (Supplementary Fig. 3) [4].

### In-silico analyses of the CAT rs1001179 SNP region

**Fig. 2:**
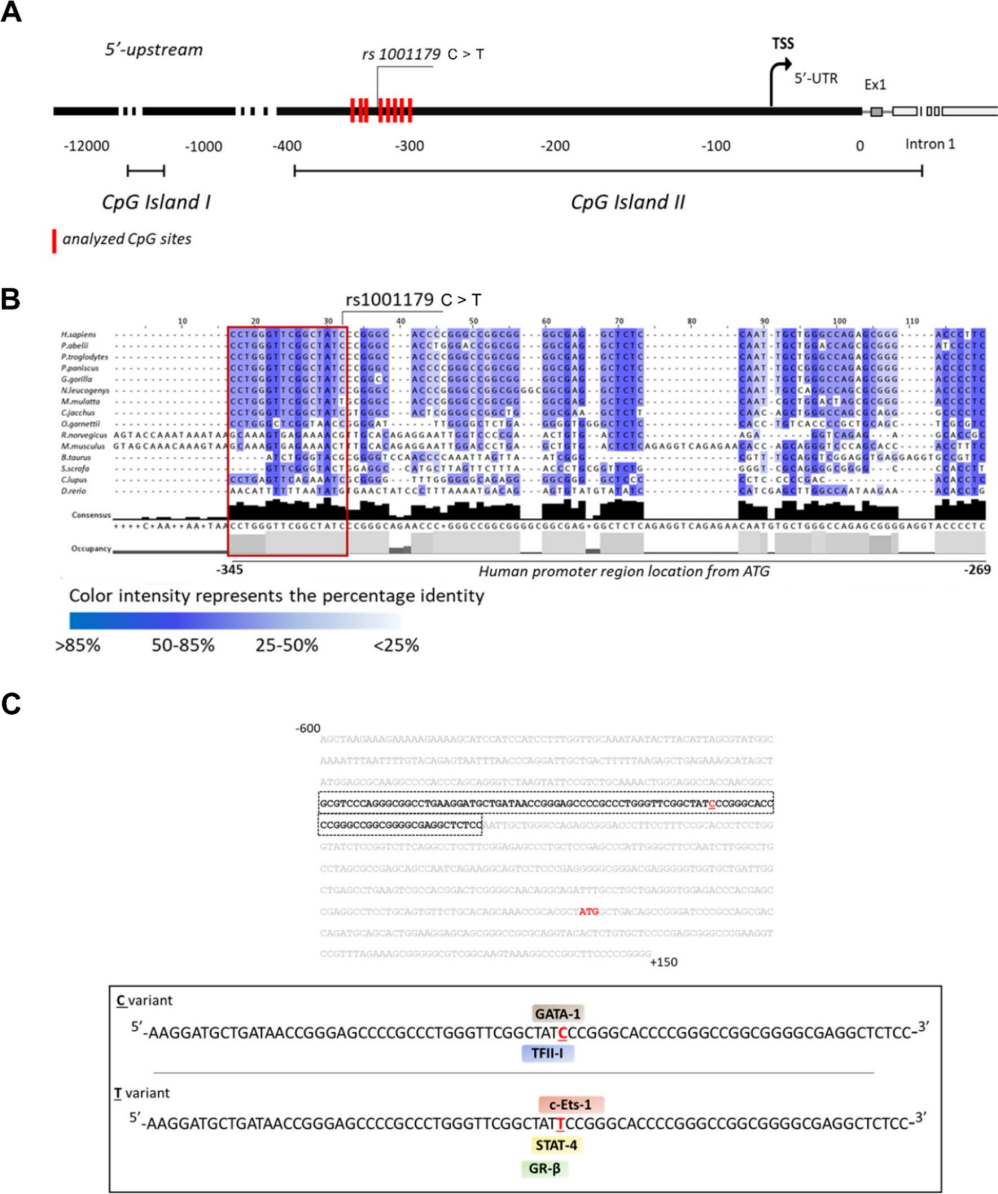
In-silico analyses of the CAT rs1001179 SNP region. **A** Schematic representation of human *CAT* promoter and location of the analyzed CpG sites. Red bars represent the location of the 8 CpG sites on the proximal region of human *CAT* promoter that have been analyzed with bisulfite conversion and pyrosequencing. The analyzed CpG sites range from ATG − 357 to − 306. This region encompasses the rs1001179 SNP. **B** Multiple sequence alignment of a selected upstream promoter region among 15 *CAT* genes belonging to primates, no-primate mammals, rodents, and zebrafish. GenBank accession references: Homo sapiens (human), NC_000011.10; Pongo abelii (orangutan), NC_036914.1; Pan troglodytes (chimpanzee), NC_036890.1; Pan paniscus (pygmychimpanzee), NC_048250.1; Gorilla gorilla (gorilla), NC_044613.1; Nomascus leucogenys (white-cheeked gibbon), NC_044395.1; Macaca mulatta (rhesus macaque), NC_041767.1; Callithrix jacchus (common marmoset), NC_048393.1; Otolemur garnettii (greatergalago), NW_003852396.1; Rattus norvegicus (brownrat), NC_005102.4; Mus musculus (house mouse), NC_000068.8; Bos taurus (cattle), NC_037342.1; Sus scrofa (wild boar), NC_010444.4; Canis lupus familiaris (dog), NC_006600.3; and Daniorerio (zebrafish), NC_007136.7. **C** Putative binding sites for TFs associated with the rs1001179 SNP C allele (upper panel) or with the rs1001179 SNP T allele (lower panel). GATA-1: GATA-binding factor 1; TFII-I: Transcription Factor II-I; STAT4: Signal Transducer and Activator of Transcription 4; c-Ets-1: ETS Proto-Oncogene 1; GR-β: Glucocorticoid Receptor beta

To investigate mechanisms underlying differential catalase expression in leukemia, among the SNPs in the human *CAT* gene we focused on the rs1001179 SNP in the *CAT* promoter (Fig. 2A) since it has been associated with altered CAT expression in normal peripheral blood cells [22, 23, 24]. To support the role of this SNP in influencing *CAT* expression, we investigated the conservation of the region in close proximity to the rs1001179 SNP (from − 345 to − 269, positions from ATG) across phylogenetically related species, using multiple sequence alignments and statistical coupling analysis. We analyzed *CAT* upstream promoter regions encompassing the rs1001179 SNP in species that include primates, non-primate mammals, rodents, and zebrafish. The analyzed sequence showed several regions with a high percentage identity among species interspersed with long insertions in rat and mouse (Fig. 2B). Remarkably, the region upstream and encompassing the rs1001179 (included in the red box of Fig. 2B; from − 345 to − 330, human *CAT* promoter region location from ATG) was highly conserved among primates, with a percentage identity of 75% (Fig. 2B).

SNPs occurring in gene regulatory sequences, such as the promoter or 5′-UTR regions, may interfere with gene expression creating or disrupting transcription factor (TF) binding sites [30]. The finding that the *CAT* rs1001179 SNP region is rich in TF binding sites [15], prompted us to investigate the possible influence of this SNP in modifying the putative TF binding sites. *In-silico* analysis of the 750 bp promoter region 600 upstream and 150 downstream of ATG (Ch11:34,438,414–34,439,164) predicted that the two alleles of the rs1001179 SNP involve changes in the TF binding sequences (Fig. 2C). In particular, the presence of C allele predicted binding sites for the General Transcription Factor II-I (TFII-I), and GATA-binding factor 1 (GATA-1). Otherwise, the presence of T allele disrupted the binding sequence for TFII-I and GATA-1 and created putative binding sites for Signal Transducer and Activator of Transcription 4 (STAT4), ETS Proto-Oncogene 1 (c-Ets-1) and Glucocorticoid Receptor beta (GR-β) (Fig. 2C).

Taken together, these data suggest that the rs1001179 SNP plays a crucial role in controlling *CAT* expression. Moreover, the in-silico prediction of TF binding sites highlights the role of rs101179 SNP in transcriptional regulation of *CAT* expression.

### The rs1001179 SNP is associated with different CAT levels

**Fig. 3:**
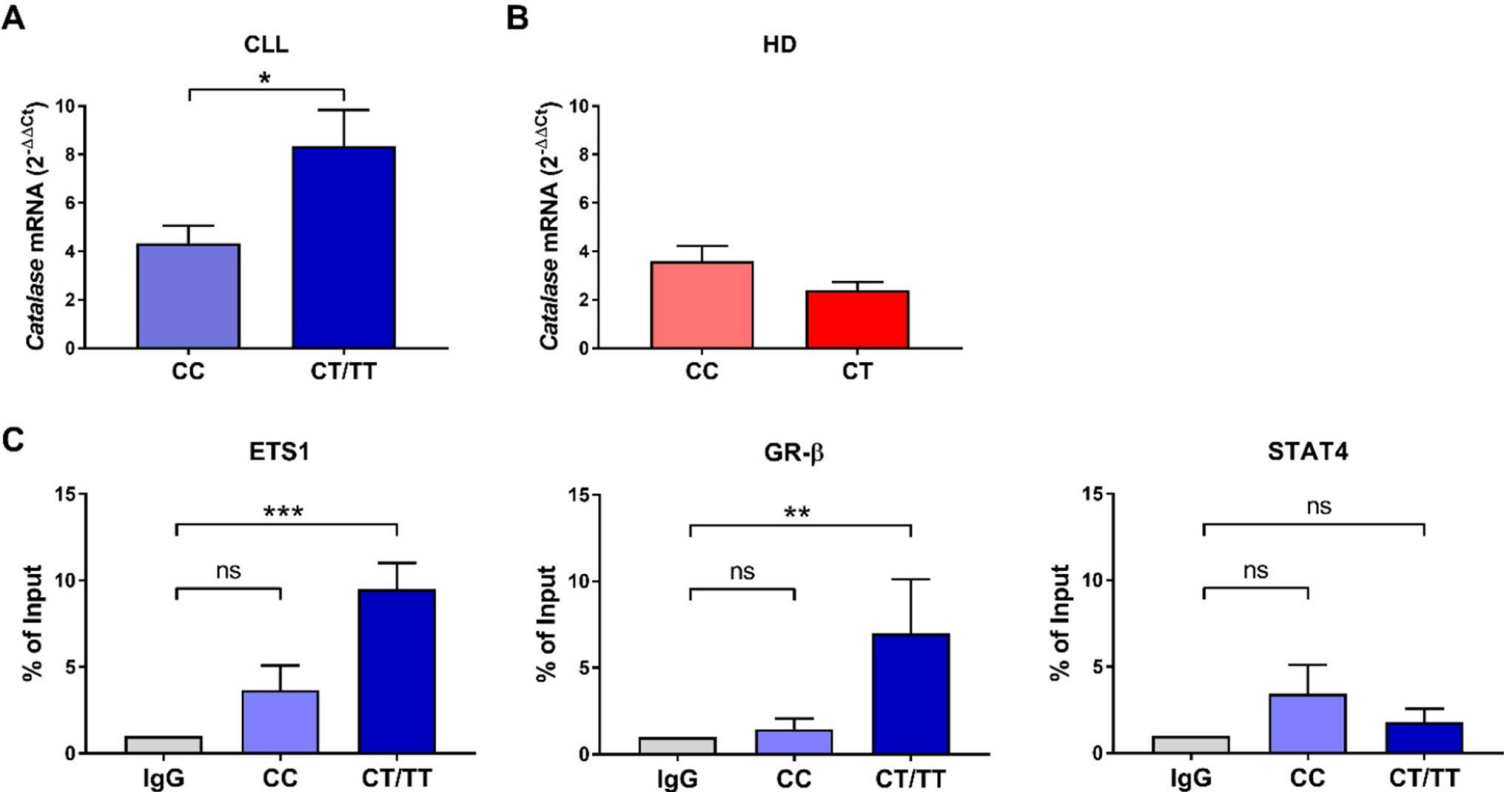
Association between *CAT* mRNA expression and rs1001179 SNP. **A** Comparison of *CAT* mRNA levels between the CC and CT/TT genotypes of rs1001179 SNP in CLL cells (*n* = 33). **B** Comparison of *CAT* mRNA expression between CC and CT genotypes for rs1001179 SNP in HD B cells (*n* = 10). Data are expressed as relative quantification using comparative Ct method (2^–ΔΔCt^), normalized to the expression value of the human embryonic kidney 293 cell line (HEK293) set as 1, and reported as mean ± SEM. Comparisons were performed with Mann Whitney test. *P < 0.05. **C** ChIP assay for TFs binding to CAT promoter in primary CLL cells harboring CC (*n* = 5) and CT/TT (*n* = 7) genotypes. Cross-linked chromatin was immunoprecipitated with antibodies against ETS1, GR-β, STAT4 or Non-Immune IgG negative control (IgG). Precipitated DNA was amplified (from − 371 to − 255, human CAT promoter region location from ATG) by qPCR. Data are expressed as percentage of input DNA (un-immunoprecipitated DNA) and reported as mean ± SEM. Comparisons were performed with Kruskal–Wallis test and each *P* value was corrected for multiple comparisons using the Dunn’s test. **P < 0.01, ***P ≤ 0.001. *CLL* chronic lymphocytic leukemia, *HDs* healthy donors, *ETS* Proto-Oncogene 1 (ETS1), Glucocorticoid Receptor beta (GR-β) and Signal Transducer and Activator of Transcription 4 (STAT4)

To investigate the role of rs1001179 SNP in controlling *CAT* expression in CLL, first we analyzed the genotype of 33 CLL patients and 10 HDs. Among the CLL patients, we detected 15 cases (45%) harboring the CC genotype, 13 patients (39%) with the CT genotype, and 5 patients (15%) with the TT genotype. The CC genotype was harbored in 5 HDs as well as the CT genotype. Then, we compared relative *CAT* mRNA levels in CLL cells grouped based on CC, CT, or TT genotypes. CLL cells harboring the TT genotype exhibited significantly higher average *CAT* mRNA levels compared with cells bearing the CC genotype whereas the CT genotype showed a trend toward association with higher *CAT* mRNA levels compared with the CC genotype (Supplementary Fig. 4). Thus, to improve the comparison statistics we grouped the CT and TT genotypes and compared *CAT* mRNA levels in CLL or HD samples between CC and CT/TT genotypes (Fig. 3). In CLL, although *CAT* mRNA levels were highly heterogeneous in both the two genotype groups (CV = 64.40% for the CC genotype; CV = 85.95% for the CT/TT genotypes), CLL cells harboring the T allele exhibited significantly higher average *CAT* mRNA levels compared with cells bearing the CC genotype (Fig. 3A). However, we failed to document an association between the rs1001179 SNP and clinical progression, measured as TTFT (Supplementary Fig. 5). In HD B cells, *CAT* mRNA levels were less heterogeneous than in CLL cells in both the genotype groups (CV = 39.43% for the CC genotype; CV = 31.01% for the T allele). *CAT* mRNA levels between the CC and CT genotype subgroups did not show significant differences (Fig. 3B).

Next, to test the ability of the TFs predicted by the bioinformatic analysis (ETS-1, GR-β and STAT4; Fig. 2C) to bind the catalase promoter in presence of the T allele, we performed ChIP assay in CLL cells harboring CC or CT/TT genotype. We compared the binding of ETS-1, GR-β and STAT4 between the two genotype groups and the Non-Immune IgG negative control (IgG). CLL cells harboring the T allele exhibited a significantly higher binding affinity for ETS-1 and GR-β than CLL cells immunoprecipitated with the IgG negative control. By contrast, CLL cells bearing the CC genotype did not show significant differences in binding affinity compared with the IgG negative control for all the analyzed TFs. In conclusion, ChIP assay data showed that CAT promoter harboring the T -but not C- allele was accessible to ETS-1 and GR-β, but not to STAT4 (Fig. 3C).

Taken together, these data indicate that genetic polymorphism may underlie, at least in part, the heterogeneous expression of *CAT* associated with variable CLL clinical behavior.

### Epigenetic regulation of CAT expression

To investigate the involvement of epigenetic regulatory mechanisms in the control of *CAT* expression, we quantified the methylation levels of 8 CpG sites within the CpG Island II of the human *CAT* gene promoter in genomic DNA from 21 CLL and 10 HD B-cell samples, using bisulfite pyrosequencing (Fig. 2A). This region encompasses the rs1001179 SNP [GRCh38 ( +)—Chr11:34,438,657–34438708] and is shown to be differentially methylated in various cell contexts, influencing *CAT* expression [26, 27]. The percentage methylation levels in each CpG site (CpG#-n) and in the overall analyzed region are shown in Table S3.

**Fig. 4:**
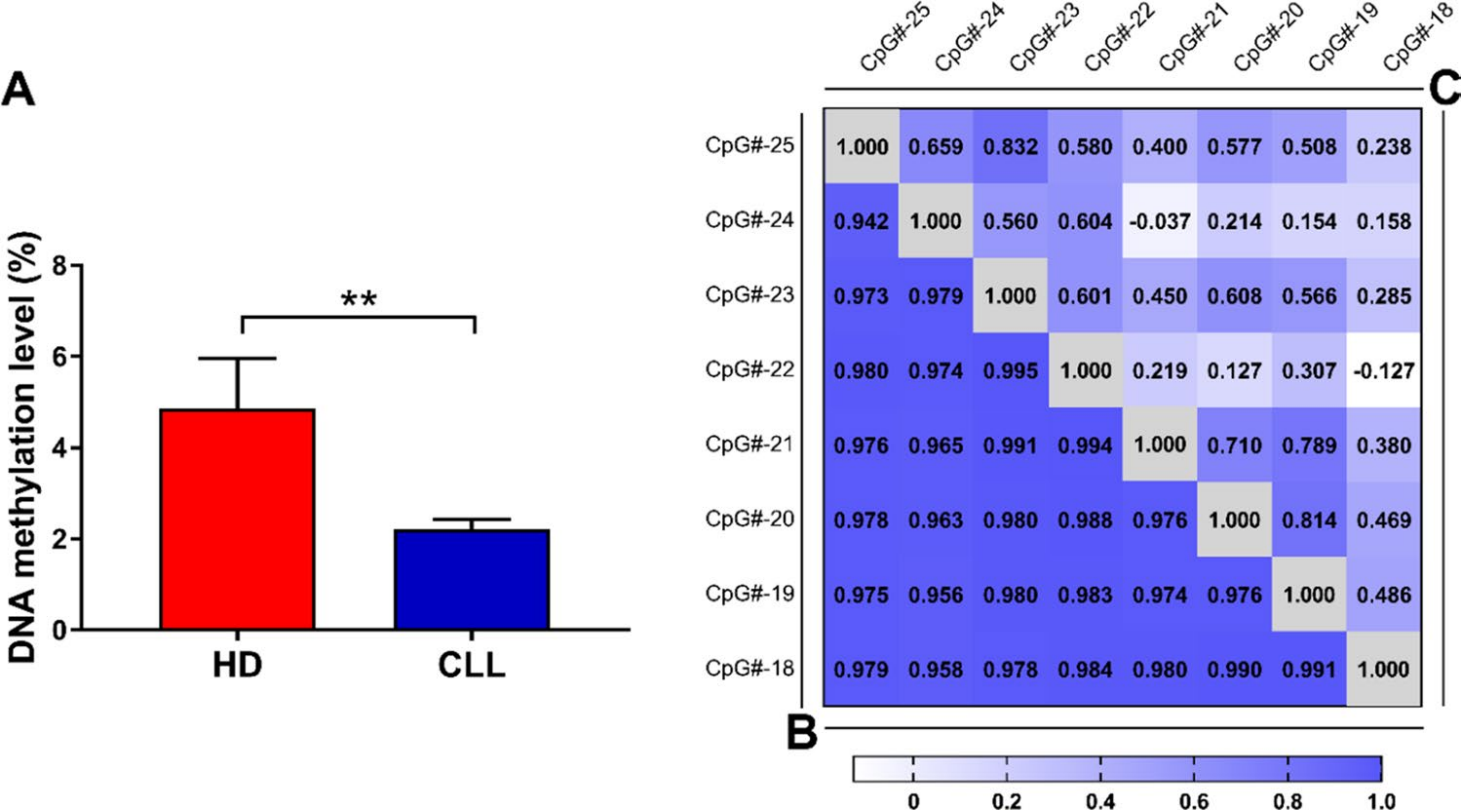
Methylation levels of *CAT* promoter region in HD and CLL B cells. **A** Methylation levels of the analyzed *CAT* promoter in HD B cells (*n* = 10) compared with CLL cells (*n* = 21). DNA methylation levels were measured as mean among the analyzed sites. Data are expressed as mean ± SEM. Comparison was performed with Student *t* test. **P < 0.01. **B** and **C** Correlation matrices showing pairwise Pearson correlations of methylation status among the 8 CpG sites within the promoter region of human *CAT* gene in HD (**B**) and CLL B cells (**C**). *HDs* healthy donors, *CLL* chronic lymphocytic leukemia

We compared the overall methylation levels between CLL and HD B cells, measured as average methylation levels of all the CpG sites, to capture the overall biologically relevant effects of methylation on gene expression. As shown in Fig. 4A, CLL cells exhibited lower methylation levels compared with HD B cells, in line with the differential *CAT* gene expression documented in those cells (Fig. 1).

Methylation is a well-regulated process and methylation levels of closer CpG sites have been shown to be correlated with each other [31, 32]. Thus, we first evaluated the correlation levels between each CpG site in HD B cells and in leukemia cells. In HD B cells, the methylation degree of CpG sites positively correlated with each other (Fig. 4B). In contrast, we observed an overall lower or even negative correlation of methylation levels among the analyzed sites in CLL cells (Fig. 4C). Therefore, CLL cells exhibit a specific methylation pattern within the *CAT* promoter, with CpG site methylation unrelated from each other, as opposed to the highly coordinated methylation observed in HD B cells.

**Fig. 5:**
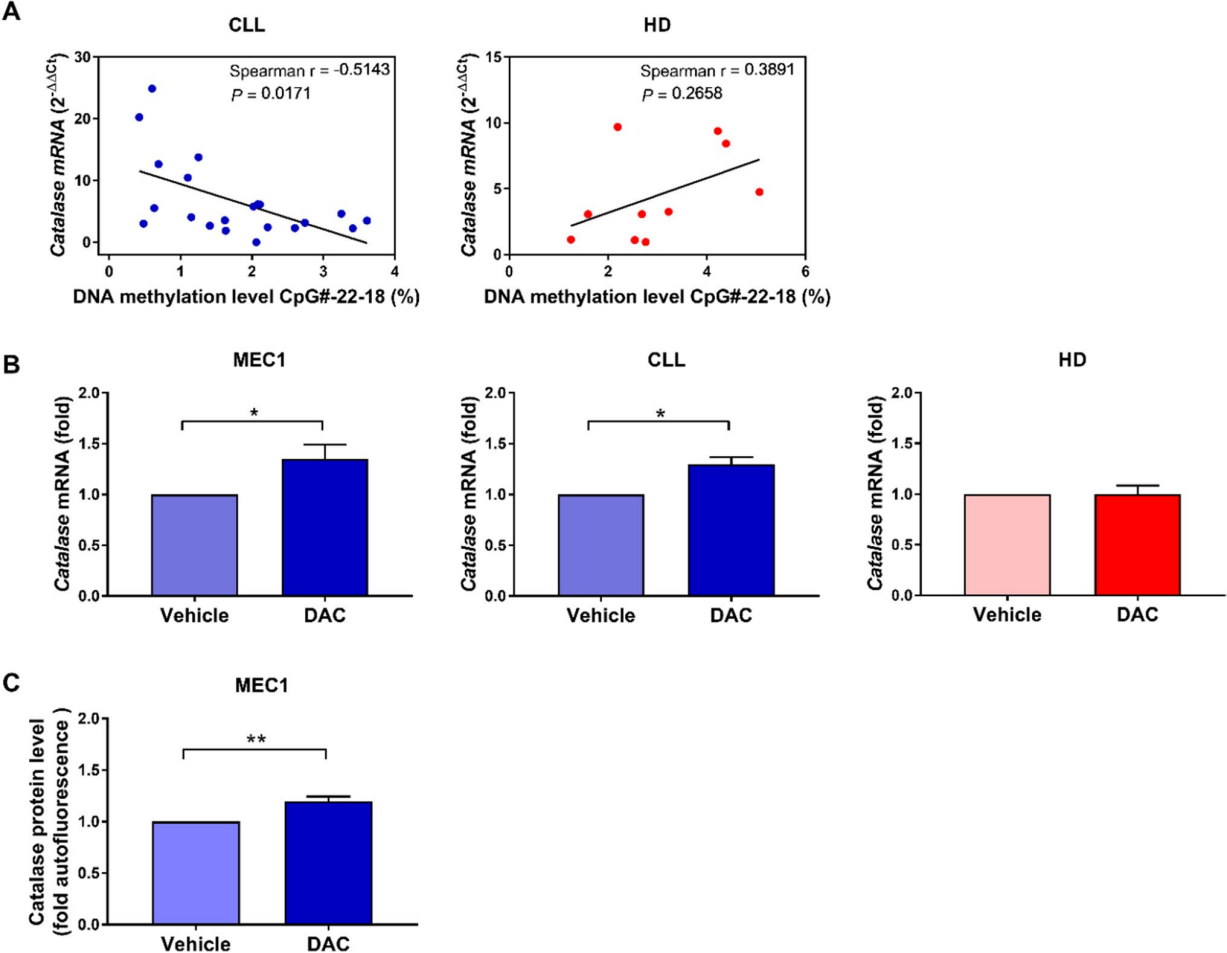
Association between methylation levels and *CAT* mRNA expression levels. **A** Association of DNA methylation percentage of sites CpG#22 to CpG#18 with *CAT* mRNA expression in CLL (*n* = 21) and HD B cells (*n* = 10). **B** MEC1 cells (6 experimental replicates), primary CLL cells (*n* = 7; CC = 4, CT = 2, ND = 1), and HD B cells (*n* = 3) were treated with 2 μM DAC or left untreated (vehicle) for 96 h and then analyzed for *CAT* mRNA levels. Data are expressed as relative quantification using comparative Ct method (2^–ΔΔCt^), normalized to the expression value of the human embryonic kidney 293 cell line (HEK293) set as 1, and reported as mean ± SEM of 5 independent experiments. **C** MEC1 cells were treated with 2 μM DAC or left untreated (vehicle) for 96 h and then analyzed for CAT protein levels. Data are expressed as fold autofluorescence calculated as median fluorescence intensity (MFI) divided by fluorescence-minus-one (FMO) and reported as mean ± SEM of 9 independent experiments. Comparisons were performed with Wilcoxon matched-pairs signed rank test. *P < 0.05. *CLL* chronic lymphocytic leukemia, *HDs* healthy donors, *DAC* 5-aza-2’-deoxycytidine, *ND* not defined for rs1001179 SNP

Further analysis showed a significant inverse relationship between the level of CpG methylation in sites CpG#22 to CpG#18 (CpG#-22-18) of the *CAT* promoter and its mRNA levels in CLL cells but not in HD B cells (Fig. 5A). Supplementary Fig. 6 shows the association of DNA methylation percentage with *CAT* mRNA expression in CLL for each site, from CpG-#22 to CpG-#18. These data suggest that methylation of the CpG Island II of the human *CAT* gene promoter regulates *CAT* expression in CLL cells. To functionally validate if the *CAT* promoter methylation plays a functional role in regulating its transcription, we analyzed *CAT* mRNA levels in MEC1, primary CLL cells, and HD B cells after treatment with the DNA methyltransferase inhibitor 5-aza-2’-deoxycytidine (DAC). As shown in Fig. 5B, inhibition of methyltransferase activity induced a significant increase of CAT mRNA in the CLL cell line MEC1 and in primary CLL cells but not in HD cells. Moreover, the DAC-induced increase of CAT was confirmed also at the protein level in MEC1 cells (Fig. 5C).

Taken together, these data show that epigenetics can regulate *CAT* expression in CLL cells via promoter methylation of the CpG Island II.

**Fig. 6:**
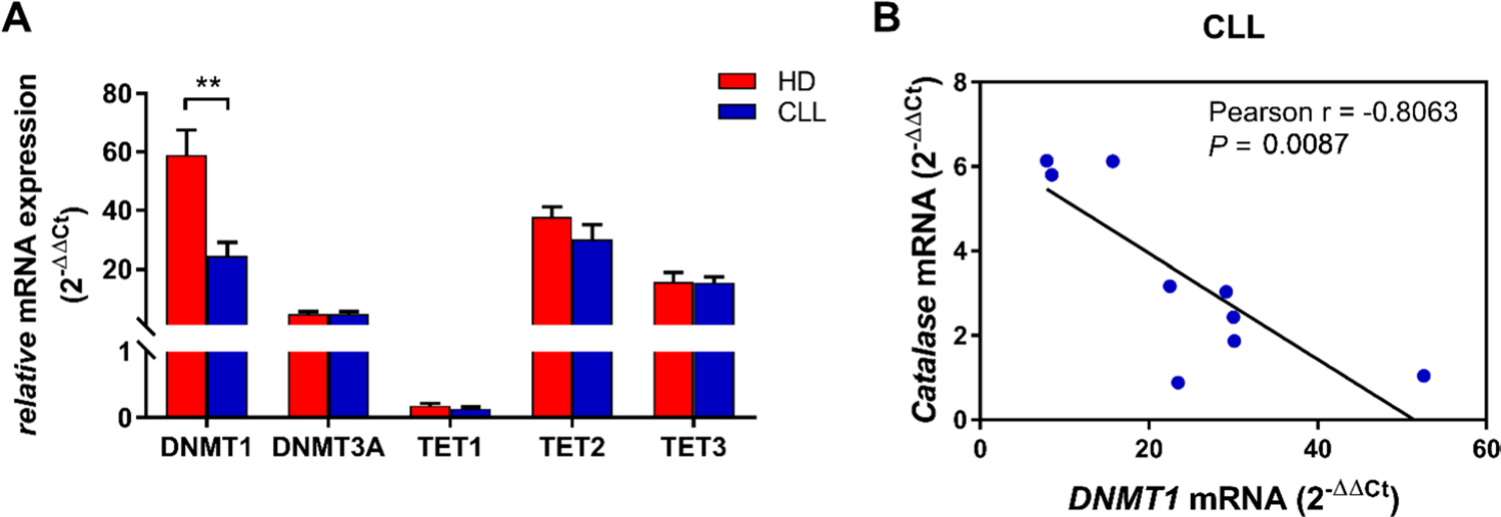
*DNMTs* and *TETs* expression levels in HD and CLL B cells. **A** *DNMTs* and *TETs* mRNA levels in HD B cells (*n* = 9) compared with CLL samples (*n* = 9). Data are expressed as relative quantification using comparative Ct method (2^–ΔΔCt^) and normalized to the expression value of the human embryonic kidney 293 cell line (HEK293) set as 1. Data are reported as mean ± SEM. Comparisons were performed with Unpaired *t* test. **P < 0.01. **B** *DNMT1* mRNA expression correlated to *CAT* mRNA expression (*n* = 9). *DNMTs* DNA methyl transferases 1, *TETs* ten-eleven translocation, *HD* healthy donor, *CLL* chronic lymphocytic leukemia

Methylation is catalyzed by several DNA methyltransferases (DNMTs) and inhibited by DNA demethylases, namely ten-eleven translocation (TET) methylcytosine dioxygenases. To assess the role of these enzymes in the methylation of CAT promoter in leukemia cells, first we characterized DNMT1, DNMT3A, TET1-3 mRNA expression levels in CLL and HD B cells (Fig. 6A). Among the analyzed methyltransferases and demethylases, expression of DNMT1 resulted significantly reduced in CLL cells compared with HD B cells (Fig. 6A), in accordance with the lower methylation levels within the CAT promoter showed by CLL versus HD B cells (Fig. 4C). Moreover, DNMT1 expression level inversely correlated with CAT expression in CLL, thus suggesting that differences in methylation levels underlying catalase expression are driven by the DNMT1 enzyme (Fig. 6B).

### Interaction of genetic and epigenetic mechanisms in regulating CAT gene expression

**Fig. 7:**
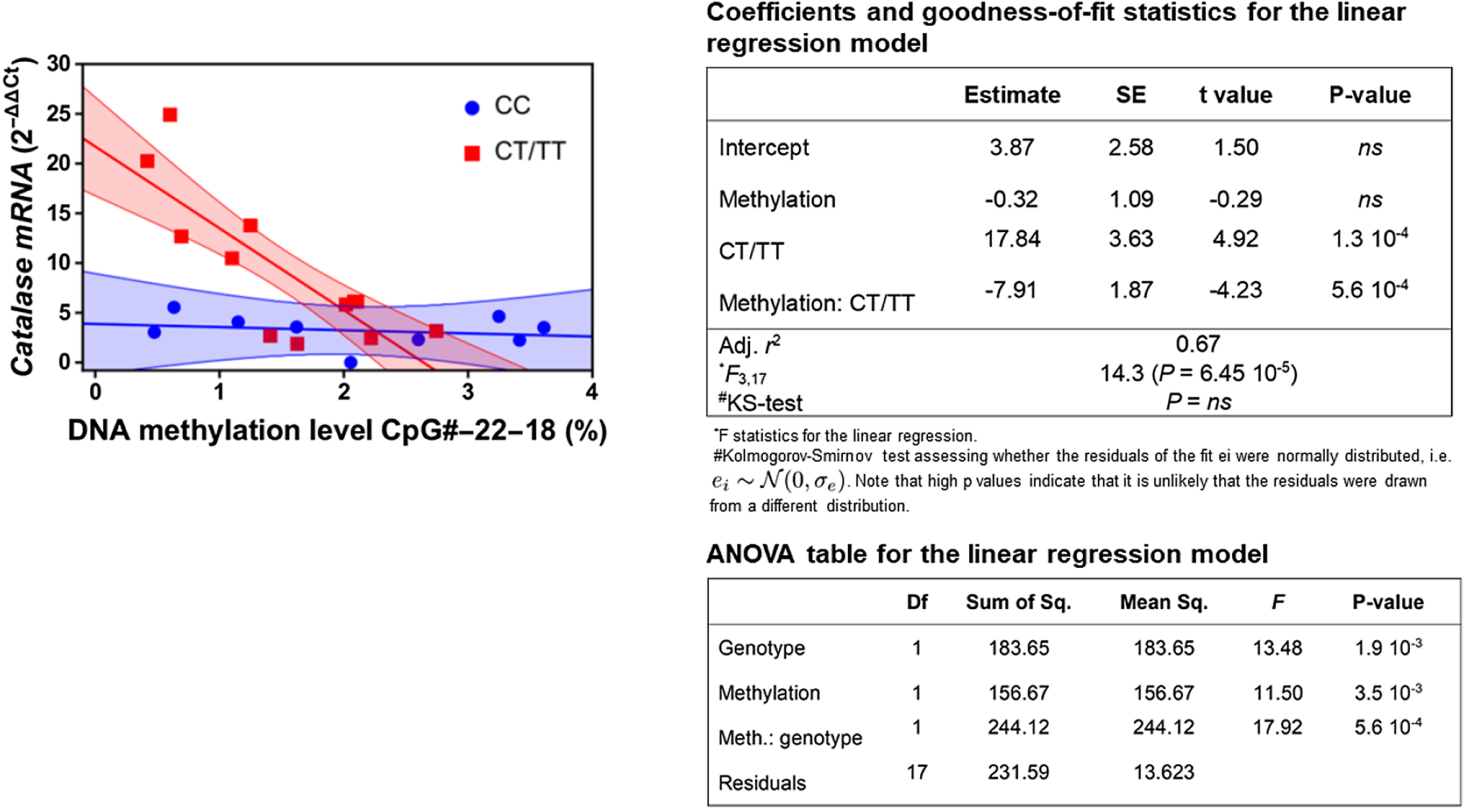
Interaction between *CAT* promoter genotype and methylation levels on catalase mRNA. The interaction has been investigated within the context of linear models. Significant regression results were found when methylation from site CpG#22 to site CpG#18 was averaged and stratified for the two genotypes CC and CT/TT. The line shows the marginal effects (i.e., predicted values) for the significant interaction between genotype CT/TT and methylation on mRNA expression. Shaded colored areas indicate the 95% confidence intervals for all interactions. Measurements stratified by genotypes are shown as points

Given that the rs1001179 SNP and methylation of the *CAT* promoter region encompassing this SNP influence *CAT* expression in CLL cells, we hypothesized that differential methylation of the promoter and the rs1001179 SNP could interact in regulating *CAT* expression. We statistically tested this hypothesis using linear models where *CAT* mRNA level was assumed to depend upon the different genotypes—a factor variable with 3 levels, the “CC”, “CT”, and “TT”—and the methylation levels—a continuous covariate—and on their interaction thereof. As shown in Supplementary Fig. 7, the interaction between methylation and CT genotype on catalase mRNA expression is statistically significant whereas a trend—although not statistically significant—towards interaction was shown between methylation and the TT genotype. The lack of statistical significance for the TT genotype could be due to the limited number of available data used to feed the model, causing predictions to be estimated with high uncertainty (Supplementary Fig. 7). Moreover, the marginal effects for the interaction between methylation and either genotypes CT and TT are quite similar and different from that estimated for genotype CC (Supplementary Fig. 7), thus indicating that the T allele is sufficient to determine the negative interaction between methylation and genotype on catalase mRNA expression. Therefore, we aggregated the CT and TT genotypes to improve regression statistics. As shown in Fig. 7, we found a significant inverse linear relationship between mean percent methylation across sites CpG#22-CpG#18 and *CAT* mRNA levels in CLL cells harboring the CT/TT genotypes.

Taken together, these data show that the CT/TT genotypes are associated with lower methylation levels and higher *CAT* expression and suggest that the rs1001179 T allele and methylation may reciprocally cooperate in regulating *CAT* expression in CLL.

## Discussion

We have recently shown that lower *CAT* expression identifies CLL patients with an indolent clinical course while higher *CAT* levels are associated with an aggressive disease [19]. In this study, we show that the rs1001179 SNP T allele in the *CAT* promoter is associated with higher *CAT* levels in CLL cells and provides binding sequences for ETS-1 and GR-β transcription factors. Moreover, methylation of the CpG Island II in the *CAT* promoter, likely driven by the DNMT1 enzyme, is a further crucial element in the regulation of *CAT* expression in CLL. Remarkably, statistical linear models suggest that the rs1001179 T allele and *CAT* promoter methylation cooperate in regulating *CAT* expression. The key advance of this study is to identify genetic and epigenetic mechanisms at the basis of the differential expression of *CAT* in CLL subsets.

Herein, we show that CLL cells express higher CAT mRNA and protein levels than normal B cells, thus confirming and extending previous data [10]. Moreover, we document that higher mRNA and protein CAT expression identifies a subset of treatment-naïve patients with a faster disease progression, thus validating our previous findings in an independent set of patients and extending the results also at the protein level. In the patient set characterized in this study, we could not find a significant association between catalase expression and the *IGHV* mutational status, in contrast with our previous results from an independent CLL patient set [19]. This finding could be explained by an intragroup CAT heterogeneity correlated with clinical outcome in otherwise molecularly homogeneous CLL groups. Differential CAT expression in CLL supports the existence of two main disease subtypes characterized by a disparity in clinical outcome, probably as a consequence of differences not only in underlying genetic lesions, epigenetic changes, activated signaling pathways, and interactions with the microenvironment, but also in the redox machinery. Therefore, the elucidation of mechanisms regulating CAT expression in CLL is of preeminent importance to unveil mechanisms of disease and to develop strategies for improving its clinical management. In this study, we focus on the rs1001179 SNP in the *CAT* promoter, since it is associated with altered CAT expression [22, 23, 24]. In-silico alignment sequence analysis of the region in close proximity to the rs1001179 SNP shows several conserved sequences among phylogenetically related species, with a higher percentage identity among primates, suggesting that this region plays a fundamental role in the *CAT* gene expression regulation. In line with the putative functional role, CLL cells harboring the rs1001179 SNP T allele exhibit higher average *CAT* mRNA levels compared with cells bearing the wild-type C allele. This finding is in accordance with previous studies showing an association between the rs1001179 SNP T allele and higher CAT levels in normal peripheral blood cells [22, 23, 24]. Moreover, a possible correlation between the rs1001179 SNP in the *CAT* promoter and susceptibility to disease has been suggested in prostate cancer and hepatocellular carcinoma [33, 34, 35]. In contrast, the rs1001179 SNP is not a risk factor for non-Hodgkin lymphoma development [36]. Taken together, these data point to genetic polymorphism as a possible mechanism underlying the heterogeneous expression of CAT associated with variable CLL clinical behavior. However, we do not document an association between the rs1001179 SNP and clinical progression, measured as TTFT. This finding could be explained by the multifactorial pattern of CAT expression regulation in cancer, which include not only genetic but also epigenetic changes and transcriptional regulation [15, 37]. Further studies on a larger patients’ set are required to address the impact of the rs1001179 SNP on CLL.

The in-silico prediction of TF-binding sites indicates that the rs1001179 SNP in the *CAT* promoter lies on a putative consensus sequence for specific TFs involved in the regulation of *CAT* expression. This analysis predicts a putative binding sequence for TFII-1 and GATA-1 in presence of the C allele, and for STAT4, ETS1 and GR-β in presence of the T allele. While previous *in-silico* analyses have already predicted the binding of GATA-1 and TFII-1 to the rs101179 SNP C allele [22, 38], and of STAT4 to the rs101179 SNP T allele [38], the putative binding of GR-β and ETS1 to rs101179 SNP has never been predicted so far. In this study, we validate the binding of GR-β and ETS-1 to the CAT promoter harboring the T -but not the C- allele. GRs can either directly bind canonical GC response elements (GREs) or act through indirect "tethered" interaction with other TFs, mediating transactivation or transrepression [39]. Moreover, several ChIP-seq studies also showed that GR can bind sequences that differ from canonical binding sequences, directly or indirectly, via other TFs [39, 40, 41]. Taken together, these data suggest that GR-β could directly bind the *CAT* promoter bearing the T allele, thus competing with ETS-1 or, alternatively, it can indirectly bind the promoter through a "tethered" interaction with ETS-1. GR-transcriptional programs exert effects on apoptosis, metabolism, and inflammation, often in collaboration with other TFs [42, 43, 44]. ETS1 is the major extracellular signal-regulated kinase 1/2 (ERK1/2) downstream effector [45, 46]. Interestingly, higher ERK1/2 activation identifies CLL patients with a faster disease progression [47, 48]. The findings that CLL patients with more aggressive disease are characterized by higher *CAT* levels [19] and ERK1/2 activation [19, 49], together with data on the function of rs1001179 T as a binding sequence for ETS1, could be suggestive of a possible role of the ERK1/2-ETS1 pathway in the transcriptional regulation of *CAT* that deserves to be further investigated.

This study also shows that CLL cells exhibit lower *CAT* promoter methylation compared with normal B cells, which could reflect the massive DNA hypomethylation that characterize CLL cells [50]. Moreover, while in normal B cells the methylation degree of CpG sites positively correlated with each other, in CLL cells we show an overall lower or even negative correlation of methylation levels among the CpG analyzed sites. Overall, methylation has been described as a well-regulated, non-random process throughout the genome and, based on this regulated process, closer neighboring CpG sites are more likely to share the same methylation status [31]. Thus, this leukemia-specific methylation pattern suggests that the co-methylation process between nearby CpG sites may be dysregulated in the *CAT* promoter of CLL cells. Moreover, methylation of the CpG Island II of the human *CAT* gene promoter negatively correlates with *CAT* mRNA levels. Remarkably, inhibition of DNA methyltransferase in CLL cells induces an augment of *CAT* mRNA levels, thus functionally validating the role of methylation in regulating *CAT* gene expression in CLL. However, DNA-methyltransferase inhibition does not completely restore *CAT* expression, thus suggesting that other mechanisms beside methylation are involved in the regulation of *CAT* expression in CLL, in line with the multifactorial nature of CAT expression regulation in cancer [15].

The expression of *DNMT1* resulted significantly reduced in CLL cells compared with HD B cells, reflecting the lower methylation levels within the CAT promoter shown by CLL versus HD B cells. In addition, *DNMT1* expression level inversely correlated with *CAT* expression in CLL, highlighting its role in modulating methylation of the CpG Island II in the CAT promoter. Therefore, these results identify DNMT1 as a driver of differences in methylation levels underlying catalase expression.

Using statistical linear models, we show that CLL cells carrying the rs1001179 SNP T allele also exhibit a lower CpG Island II methylation in the *CAT* promoter and a higher *CAT* expression. This finding suggests that methylation of the promoter region encompassing the rs1001179 SNP could modify the effects of this SNP on *CAT* expression in leukemia cells, for example influencing the binding affinity of TFs to DNA sites, as reported for other genes [51, 52, 53]. Indeed, some transcription factors preferably bind hypermethylated DNA while others are inhibited by hypermethylated CpG sites [54]. Herein, we also show that ETS-1 can bind the *CAT* promoter in presence of rs1001179 SNP T allele, which in turn results associated with higher CAT levels in CLL cells but not in HD B cells. Interestingly, DNA binding of ETS-1 is known to preferably bind hypomethylated DNA [54]. Taken together, these data could account for the finding that rs1001179 SNP does not influence CAT expression in HD B cells, which are indeed characterized by higher *CAT* promoter methylation levels, compared with leukemic cells. Remarkably, SNPs can also influence the methylation status of surrounding CpG sites operating as a cis-acting factor for methylation of adjacent CpG sites [30, 55]. Therefore, the potential interactions of these regulatory mechanisms can alter the binding of TFs to DNA in an allele-specific manner, thus playing a role in disease risk and cancer progression. Also, targeting CAT regulatory pathways may be an interesting therapeutic strategy to be used in combination with the existing ones, with the aim to overcome drug resistance in CLL. Interestingly, potential catalase inhibitors in cancer are being investigated [15, 56, 57, 58, 59, 60] whilst there is very scanty evidence on CAT regulatory pathways in relation to drug resistance [61]. However, further investigations are required to address the impact of genetic and epigenetic mechanisms of catalase regulation as well as their interactions on leukemia progression and resistance.

In conclusion, our data advance the knowledge of the role of genetic and epigenetic mechanisms controlling *CAT* expression in leukemia. Future challenges are to design therapeutics strategies targeting CAT regulatory pathways that could implement the effectiveness of current therapies and overcome drug resistance in CLL.

## Supplementary Information

Below is the link to the electronic supplementary material.

Supplementary file1 (DOCX 53 KB)

Supplementary file2 (DOCX 2661 KB)

## Data Availability

The data used and/or analyzed during the current study are available from the corresponding author on reasonable request.
