# Supplementary material for: The rs1001179 SNP and CpG methylation regulate catalase expression in chronic lymphocytic leukemia
Source: Cell Mol Life Sci. 2022 Sep 16;79(10):521. doi: 10.1007/s00018-022-04540-7 (PMC9481481; doi:10.1007/s00018-022-04540-7)
Supplement: Supplementary file 1 — Supplementary file1 (DOCX 53 KB) [file 18_2022_4540_MOESM1_ESM.docx]

**The rs1001179 SNP and CpG methylation regulate catalase expression in chronic lymphocytic leukemia**

Marilisa Galasso ^a,b^, Elisa Dalla Pozza ^a^, Roberto Chignola ^c^, Simona Gambino ^a^, Chiara Cavallini ^d^, Francesca Maria Quaglia ^b^, Ornella Lovato ^d^, Ilaria Dando ^a^, Giorgio Malpeli ^e^, Mauro Krampera ^b^, Massimo Donadelli ^a^, Maria G. Romanelli ^a*^, Maria T. Scupoli ^a,d*^.

*^a^Department of Neurosciences, Biomedicine and Movement Sciences, University of Verona, Strada Le Grazie 8, 37134, Verona, Italy.*

*^b^Department of Medicine, Section of Hematology, University of Verona, Policlinico G.B. Rossi, P. L.A. Scuro 10, 37134, Verona, Italy.*

*^c^Department of Biotechnology, University of Verona, Strada Le Grazie 15, 37134 Verona, Italy.*

*^d^Research Center LURM, Interdepartmental Laboratory of Medical Research, University of Verona, Policlinico G.B. Rossi, P. L.A. Scuro 10, 37134, Verona, Italy.*

*^e^Department of Surgery, Dentistry, Pediatrics, and Gynecology, University of Verona, Verona, Policlinico G.B. Rossi, P. L.A. Scuro 10, 37134, Italy.*

*^*^*Corresponding authors.

**Corresponding authors:**

Maria T. Scupoli, PhD, Laboratorio Universitario di Ricerca Medica (LURM), Policlinico G.B. Rossi, P. L.A. Scuro 10, 37134 Verona, Italy; Phone: +39-045-812-8425, Fax: +39-045-802-7403; e-mail: [mariateresa.scupoli@univr.it](mailto:mariateresa.scupoli@univr.it)

Maria G. Romanelli, Department of Neurosciences, Biomedicine and Movement Sciences, Biology and Genetics Section, University of Verona, Strada Le Grazie 8, 37134 Verona, Italy; Phone: +39-045-802- 7182, Fax: +39-045-802-7180, e-mail: [mariagrazia.romanelli@univr.it](mailto:mariateresa.scupoli@univr.it)

**Supplementary Information**

**Supplementary Methods**

***Sample preparation***

Peripheral blood mononuclear cells (PBMCs) were isolated by Ficoll-hypaque (Lymphoprep; Nicomed, NO, EU) centrifugation and stored in liquid nitrogen. Upon thawing, only samples with at least 85% viability, assessed using 7-Amino-Actinomycin (7-AAD) dye (BD Biosciences, CA, USA) and flow cytometry (FACSCanto; Becton Dickinson, NJ, USA), were processed further. For quantitative polymerase chain reaction (qPCR) and pyrosequencing, CLL cells from samples with <70% B cells were isolated by negative selection using Human B-Cell Enrichment Kit (Stem Cell Technologies, BC, CA). After separation, cell purity was routinely above 98%, as assessed with CD19 staining and flow cytometry (FACSCanto). B cells from healthy-donor PBMCs were isolated using flow cytometry activated cell sorting (FACS) (FACSAria, Becton Dickinson) using CD19-PerCpCy5.5 antibody (BD Biosciences) and purity mask. B cell purity post sorting was above 98%, as assessed with CD19 staining and flow cytometry (FACSAria).

***Quantitative reverse transcription polymerase chain reaction***

Total RNA was extracted using TRIzol Reagent (Thermo Fisher Scientific, MA, USA) and 1 µg of RNA was reverse transcribed using M-MLV Reverse Transcriptase (Invitrogen, CA, USA). For m*RNA* quantification the primers were: *Catalase* F, 5’-GAACTGTCCCTACCGTGCTCGA-3’; *Catalase* R, 5’-CCAGAATATTGGATGCTGTGCTCCAGG-3’; *DNMT1* F, 5’-TACCTGGACGACCCTGACCTC-3’; *DNMT1* R, 5’- CGTTGGCATCAAAGATGGACA-3’; *DNMT3A* F, 5’-TATTGATGAGCGCACAAGAGAGC-3’; *DNMT3A* R, 5’-GGGTGTTCCAGGGTAACATTGAG-3’; TET1 F, 5’- ATACAATGGGCACCCTACCG-3’; *TET1* R, 5’-GGGCTTGGGCTTCTACCAAA-3’; TET2 F, 5’- GATAGAACCAACCATGTTGAGGG-3’; *TET2* R, 5’-TGGAGCTTTGTAGCCAGAGGT-3’; TET3 F, 5’- TACCAACCGCCGCACGCAC -3’; *TET3* R, 5’- AGCCGCTCCTTGTCCCCAC -3’ . Normalization was performed analyzing the ribosomal protein large P0 (RPLP0) mRNA expression level, using the following primers: RPLP0 F, 5'-ACATGTTGCTGGCCAATAAGGT-3' and RPLP0 R, 5'-CCTAAAGCCTGGAAAAAGGAGG-3'. The thermal cycle reaction was performed as follows: 95°C for 10 min followed by 40 cycles at 95°C for 15 sec and 60°C for 1 min. The average of cycle threshold of each triplicate was analyzed according to the 2^‑ΔΔCt^ method. Human embryonic kidney 293 cell line (HEK293) was used as calibrator sample to normalize the expression values of the samples.

***Flow cytometry***

PBMCs from healthy donors were stained with CD19-BV786 and CD3/CD14-APCCy7. PBMCs from CLL patients were stained with CD5-BV605, CD19-BV786, and CD3/CD14-APCCy7 (Table S2). After incubation with antibodies for 15 min at room temperature (rt) in the dark, cells were washed, fixed with BD Phosflow™ Fix Buffer I (BD Biosciences) and permeabilized with PBS (1X) + 0,1% TritonX-100 for 15 min rt in the dark. Permeabilized cells were washed, pelleted, and stained with anti-cleaved poly ADP-ribose polymerase (c-PARP)-Alexa Fluor647 and anti-catalase-Alexa Fluor 488 (Table S2). For catalase measurement, approximately 2.0x10^4^ gated events were acquired for each sample on a BD LSRFortessa flow cytometer (Becton Dickinson). Flow cytometry data were processed using FlowJo software (v10 TreeStar, OR, USA). For data analysis, identification of healthy and leukemic B cells was based on CD19 expression and CD5/CD19 co-expression, respectively. Debris were excluded based on forward-scatter and side-scatter and residual T and monocytes cells were excluded based on CD3 and CD14 signals, respectively. Viable cells were defined as c-PARP negative cells.

***Software and Statistical analysis***

Multiple sequence alignment was conducted using online MUSCLE software (https://www.ebi.ac.uk/Tools/msa/muscle/). Jalview software was used to calculate the percent identity among sequences. (v. 2.11.1.3, Jalview Software Barton Group, University of Dundee, Scotland, UK). PROMO online software, version 8.3 of TRANSFAC, was used for prediction of transcriptional factor-binding sites (<http://alggen.lsi.upc.es/cgi-bin/promo_v3/promo/promoinit.cgi?dirDB=TF_8.3>). Linear regression analyses were carried out using the open-source platform for statistical computing R (version 3.6.0) run under the free integrated development environment RStudio (version 1.0.153, https://rstudio.com). We developed several models to study the interactions between catalase-promoter genotypes and methylation levels in association with catalase mRNA levels in CLL patients [1]. The goodness of the different fits was determined by ANOVA. The R package *ggeffects* [2] was also used to compute the marginal effects, i.e. the mean response of the factor variable (i.e. genotype) adjusted for the covariate (methylation levels), and the corresponding 95% confidence intervals.

***Supplemental Tables***

**Table S1.** **Clinical and biological characteristics of CLL patients**

|  | n=75 |
| --- | --- |
| *Gender*  Male  Female | 47 (62.7%)  28 (37.3%) |
| **Age at the diagnosis (years)*  Median (range) | 66 (36-92) |
| *^#^TTFT (months)*  Median (range) | 64.6 (1.1-306.7) |
| *Binet*  Binet A  Binet B  Binet C  NA | 56 (74.6%)  15 (20.0%)  2 (2.7%)  2 (2.7%) |
| *^$^CD38*  Negative  Positive  NA | 19 (25.3%)  55 (73.3%)  1 (1.4%) |
| *^†^ZAP70*  Negative  Positive  NA | 20 (26.7%)  46 (61.3%)  9 (12.0%) |
| *^‡^IGHV*  UM  M  NA | 25 (33.3%)  35 (46.7%)  15 (20.0%) |
| ^§§^*Cytogenetics*  Favorable  Neutral  Unfavorable  NA | 20 (26.7%)  27 (36.0%)  13 (17.3%)  15 (20.0%) |

*CLL diagnosed according to 2008 Guidelines for Diagnosis and Treatment of CLL [3];

#TTFT: time-to-first-treatment;

$CD38 was determined using a 30% cut-off;

†ZAP70 was determined using a 20% cut-off;

‡IGHV sequencing utilized a 2% cut-off to discriminate mutated from unmutated IGHV; M: mutated; UM: unmutated;

**^§§^**Patients were stratified into major cytogenetic categories, based on NCCN CLL Guidelines [4]: favorable (del 13q as a sole aberration), neutral (normal karyotype, trisomy 12q), and unfavorable (11q and/or 17p deletion);

NA: not available.

Table S2. Antibodies used for catalase detection

| Antibody | Fluorochrome | Clone | Manufacturer |
| --- | --- | --- | --- |
| Catalase | Alexa Fluor488 | EP1929Y | Abcam |
| Cleaved PARP (cPARP) | Alexa Fluor647 | F21-852 | BD Biosciences |
| CD19 | BV786 | SJ25C1 | BD Biosciences |
| CD3 | APCCy7 | UCHT1 | BioLegend |
| CD14 | APCCy7 | M5E2 | BioLegend |
| CD5 | BV605 | UCHT2 | BD Biosciences |

**Table S3. DNA methylation of catalase promoter CpG sites in HD and CLL B cells**

| CpG sites (% Methylation) | | | | | | | | | Overall Region |
| --- | --- | --- | --- | --- | --- | --- | --- | --- | --- |
| Sample | CpG#-25 | CpG#-24 | ^*^CpG#-23 | CpG#-22 | CpG#-21 | CpG#-20 | CpG#-19 | CpG#-18 | ^#^CpG#-25-18 |
| HD#002 | 9.3 | 9.4 | 9.8 | 9.2 | 9.6 | 9.2 | 6.1 | 8.1 | 8.8 |
| HD#002 | 1.3 | 3.9 | 2.3 | 1.9 | 1.3 | 2.3 | 0.0 | 0.0 | 1.6 |
| HD#003 | 10.0 | 10.5 | 11.7 | 10.7 | 11.3 | 8.9 | 7.7 | 8.2 | 9.9 |
| HD#004 | 0.0 | 3.3 | 3.1 | 1.9 | 1.8 | 2.0 | 0.0 | 0.0 | 1.5 |
| HD#005 | 3.0 | 5.5 | 4.6 | 3.3 | 3.4 | 3.3 | 2.6 | 2.8 | 3.6 |
| HD#006 | 1.6 | 1.9 | 2.6 | 1.7 | 1.4 | 1.7 | 0.0 | 0.0 | 1.4 |
| HD#007 | 4.1 | 4.4 | 4.7 | 3.9 | 3.0 | 4.1 | 2.8 | 2.5 | 3.7 |
| HD#008 | 2.4 | 3.9 | 3.9 | 3.7 | 3.4 | 3.5 | 2.3 | 2.6 | 3.2 |
| HD#009 | 5.2 | 4.7 | 5.4 | 4.9 | 5.1 | 5.4 | 3.8 | 4.5 | 4.9 |
| HD#010 | 9.4 | 11.1 | 11.6 | 10.7 | 10.1 | 10.4 | 8.0 | 9.3 | 10.1 |
| CLL#001 | 1.5 | 3.8 | 2.6 | 1.6 | 1.6 | 0.0 | 0.0 | 0.0 | 1.4 |
| CLL#002 | 4.8 | 4.3 | 4.5 | 3.4 | 4.6 | 3.9 | 2.3 | 2.1 | 3.7 |
| CLL#003 | 4.0 | 3.4 | 4.9 | 0.0 | 4.3 | 6.4 | 2.8 | 3.7 | 3.7 |
| CLL#004 | 5.4 | 3.5 | 5.3 | 4.1 | 4.9 | 5.9 | 3.3 | 0.0 | 4.0 |
| CLL#005 | 4.6 | 3.2 | 4.4 | 2.8 | 4.1 | 4.3 | 1.9 | 0.0 | 3.1 |
| CLL#006 | 1.8 | 1.9 | 3.4 | 0.0 | 2.1 | 0.0 | 0.0 | 0.0 | 1.1 |
| CLL#007 | 4.5 | 4.0 | 3.4 | 0.0 | 0.0 | 3.0 | 0.0 | 0.0 | 1.9 |
| CLL#008 | 1.9 | 1.6 | 3.0 | 1.7 | 1.8 | 0.0 | 0.0 | 0.0 | 1.2 |
| CLL#009 | 3.7 | 3.4 | 2.9 | 3.0 | 2.6 | 0.0 | 0.0 | 0.0 | 1.9 |
| CLL#010 | 5.1 | 5.6 | 5.1 | 6.3 | 0.0 | 0.0 | 0.0 | 0.0 | 2.7 |
| CLL#011 | 2.3 | 3.8 | 4.0 | 3.1 | 2.6 | 2.7 | 1.6 | 0.0 | 2.5 |
| CLL#012 | 2.6 | 3.4 | 3.2 | 2.4 | 3.3 | 3.4 | 1.5 | 0.0 | 2.5 |
| CLL#013 | 3.1 | 3.5 | 4.8 | 3.1 | 2.9 | 2.7 | 1.7 | 0.0 | 2.7 |
| CLL#014 | 3.0 | 2.9 | 2.7 | 2.3 | 3.3 | 2.5 | 0.0 | 0.0 | 2.1 |
| CLL#015 | 2.6 | 3.1 | 3.2 | 2.6 | 2.8 | 2.5 | 1.6 | 1.7 | 2.5 |
| CLL#016 | 3.5 | 2.9 | 4.0 | 3.3 | 4.3 | 3.5 | 2.6 | 0.0 | 3.0 |
| CLL#017 | 1.6 | 2.7 | 3.5 | 2.1 | 2.1 | 2.9 | 0.0 | 0.0 | 1.9 |
| CLL#018 | 0.0 | 2.2 | 0.0 | 0.0 | 2.4 | 0.0 | 0.0 | 0.0 | 0.6 |
| CLL#019 | 2.3 | 3.0 | 3.4 | 2.7 | 2.3 | 2.0 | 1.7 | 1.6 | 2.4 |
| CLL#020 | 1.9 | 2.0 | 2.3 | 1.9 | 2.2 | 2.5 | 1.6 | 0.0 | 1.8 |
| CLL#021 | 1.7 | 3.2 | 3.2 | 1.8 | 1.7 | 2.3 | 0.0 | 0.0 | 1.7 |

*The sites highlighted in red are those encompassing the rs1001179 SNP. The site CpG#22 is the closest to rs1001179 SNP;

# Mean percent methylation across sites CpG#25-CpG#18.

**References**

1. Keith TZ (2019) Multiple regression and beyond: An introduction to multiple regression and structural equation modeling. Mult Regres Beyond An Introd to Mult Regres Struct Equ Model 1–639. https://doi.org/10.4324/9781315162348/MULTIPLE-REGRESSION-BEYOND-TIMOTHY-KEITH

2. Lüdecke D (2018) ggeffects: Tidy Data Frames of Marginal Effects from Regression Models. J Open Source Softw 3:772. https://doi.org/10.21105/joss.00772

3. Hallek M, Cheson BD, Catovsky D, et al (2008) Guidelines for the diagnosis and treatment of chronic lymphocytic leukemia: A report from the International Workshop on Chronic Lymphocytic Leukemia updating the National Cancer Institute-Working Group 1996 guidelines. Blood 111:5446–5456

4. Hallek M, Shanafelt TD, Eichhorst B (2018) Chronic lymphocytic leukaemia. Lancet 391:1524–1537. https://doi.org/10.1016/S0140-6736(18)30422-7
