# Supplementary material for: The rs1001179 SNP and CpG methylation regulate catalase expression in chronic lymphocytic leukemia
Source: Cell Mol Life Sci. 2022 Sep 16;79(10):521. doi: 10.1007/s00018-022-04540-7 (PMC9481481; doi:10.1007/s00018-022-04540-7)
Supplement: Supplementary file 2 — Supplementary file2 (DOCX 2661 KB) [file 18_2022_4540_MOESM2_ESM.docx]

**The rs1001179 SNP and CpG methylation regulate catalase expression in chronic lymphocytic leukemia**

Marilisa Galasso ^a,b^, Elisa Dalla Pozza ^a^, Roberto Chignola ^c^, Simona Gambino ^a^, Chiara Cavallini ^d^, Francesca Maria Quaglia ^b^, Ornella Lovato ^d^, Ilaria Dando ^a^, Giorgio Malpeli ^e^, Mauro Krampera ^b^, Massimo Donadelli ^a^, Maria G. Romanelli ^a*^, Maria T. Scupoli ^a,d*^.

*^a^Department of Neurosciences, Biomedicine and Movement Sciences, University of Verona, Strada Le Grazie 8, 37134, Verona, Italy.*

*^b^Department of Medicine, Section of Hematology, University of Verona, Policlinico G.B. Rossi, P. L.A. Scuro 10, 37134, Verona, Italy.*

*^c^Department of Biotechnology, University of Verona, Strada Le Grazie 15, 37134 Verona, Italy.*

*^d^Research Center LURM, Interdepartmental Laboratory of Medical Research, University of Verona, Policlinico G.B. Rossi, P. L.A. Scuro 10, 37134, Verona, Italy.*

*^e^Department of Surgery, Dentistry, Pediatrics, and Gynecology, University of Verona, Verona, Policlinico G.B. Rossi, P. L.A. Scuro 10, 37134, Italy.*

*^*^*Corresponding authors.

**Corresponding authors:**

Maria T. Scupoli, PhD, Laboratorio Universitario di Ricerca Medica (LURM), Policlinico G.B. Rossi, P. L.A. Scuro 10, 37134 Verona, Italy; Phone: +39-045-812-8425, Fax: +39-045-802-7403; e-mail: [mariateresa.scupoli@univr.it](mailto:mariateresa.scupoli@univr.it)

Maria G. Romanelli, Department of Neurosciences, Biomedicine and Movement Sciences, Biology and Genetics Section, University of Verona, Strada Le Grazie 8, 37134 Verona, Italy; Phone: +39-045-802- 7182, Fax: +39-045-802-7180, e-mail: [mariagrazia.romanelli@univr.it](mailto:mariateresa.scupoli@univr.it)

**Supplementary Figures**

**Fig.1**

**
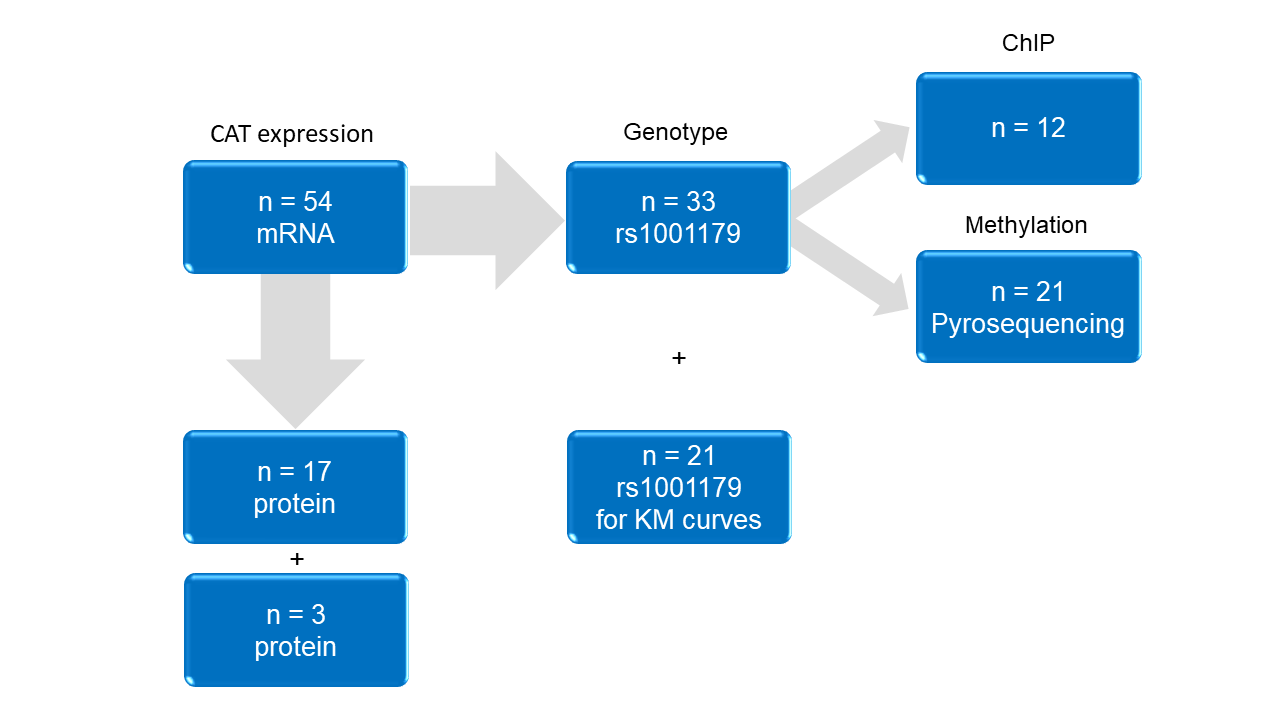
**

**Fig. 1** Workflow scheme. Arrows indicate the overlapped samples; the plus sign indicates the not overlapped samples.

***
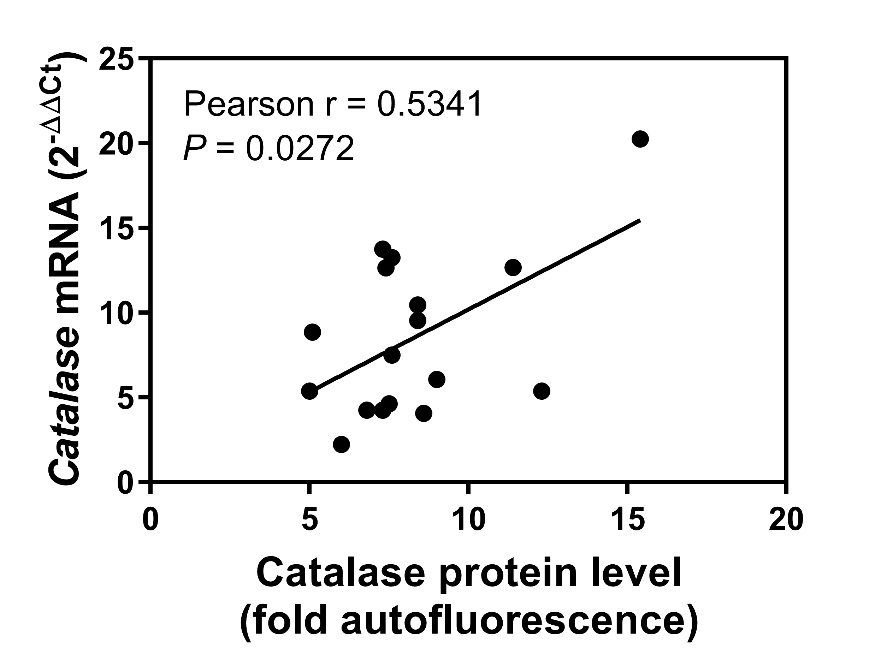
*Fig. 2**

**Fig. 2** Association between CAT mRNA and protein expression levels in CLL (n = 17).

**Fig. 3**

**
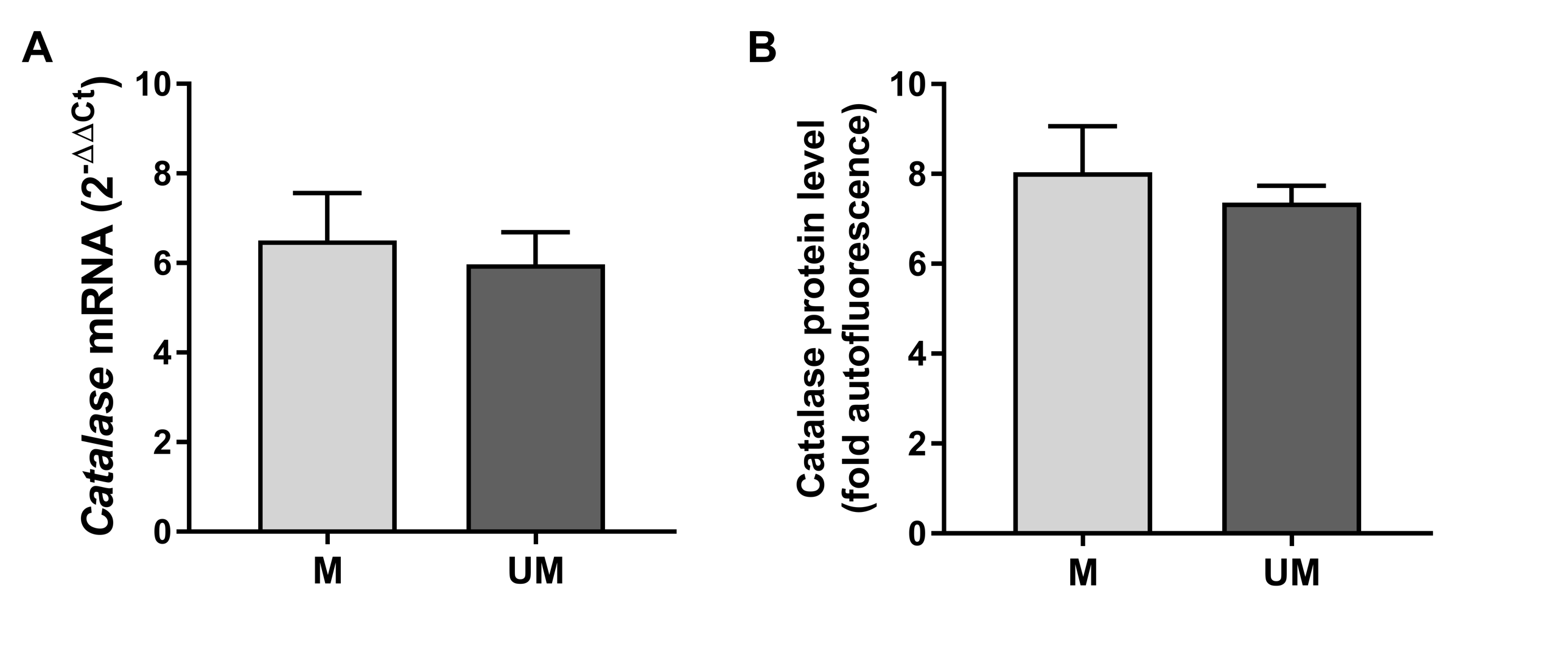
Fig. 3** Association of CAT expression levels with immunoglobulin heavy-chain variable region gene (*IGHV*) status in CLL. **(A)** CAT mRNA levels in *IGHV*-mutated (M) (n = 26) and -unmutated (UM) (n = 18) CLL. Data are expressed as relative quantification using comparative Ct method (2^–ΔΔCt^), normalized to the expression value of the human embryonic kidney 293 cell line (HEK293) set as 1. **(B)** CAT protein expression in M (n =12) and UM (n = 8) CLL. Data are expressed as fold autofluorescence calculated as median fluorescence intensity (MFI) divided by fluorescence-minus-one (FMO). *IGHV* sequencing utilized a 2% cut-off to discriminate M from UM *IGHV.* Data are reported as mean ±SEM. Comparisons were performed with Mann Whitney test.

*
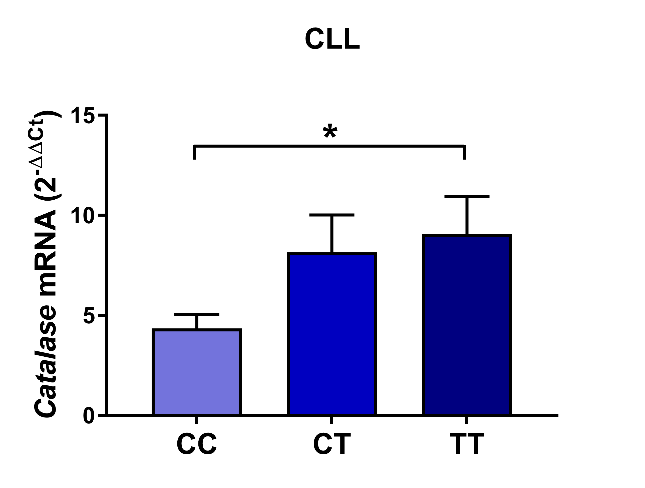
***Fig. 4**

**Fig. 4** Association between *CAT* mRNA expression and rs1001179 SNP. Comparison of *CAT* mRNA levels between the CC, (n =15) CT (n =13), and TT (n =5) genotypes of rs1001179 SNP in CLL. Data are expressed as relative quantification using comparative Ct method (2^–ΔΔCt^), normalized to the expression value of the human embryonic kidney 293 cell line (HEK293) set as 1, and reported as mean ±SEM. Comparisons were performed with Mann Whitney test. *: P < 0.05.


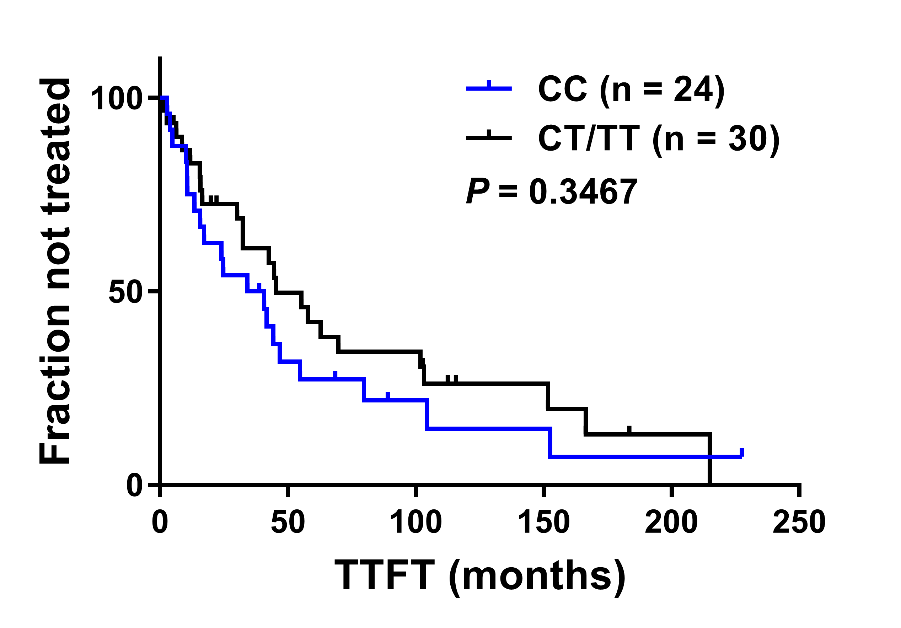
**Fig. 5**

**Fig. 5** Kaplan-Meier curves of TTFT for subgroups of CLL patients distinguished by CC (n = 24) and CT/TT (n =30) genotypes of rs1001179 SNP. Difference between the two curves was calculated with log-rank test. TTFT: time to first treatment.


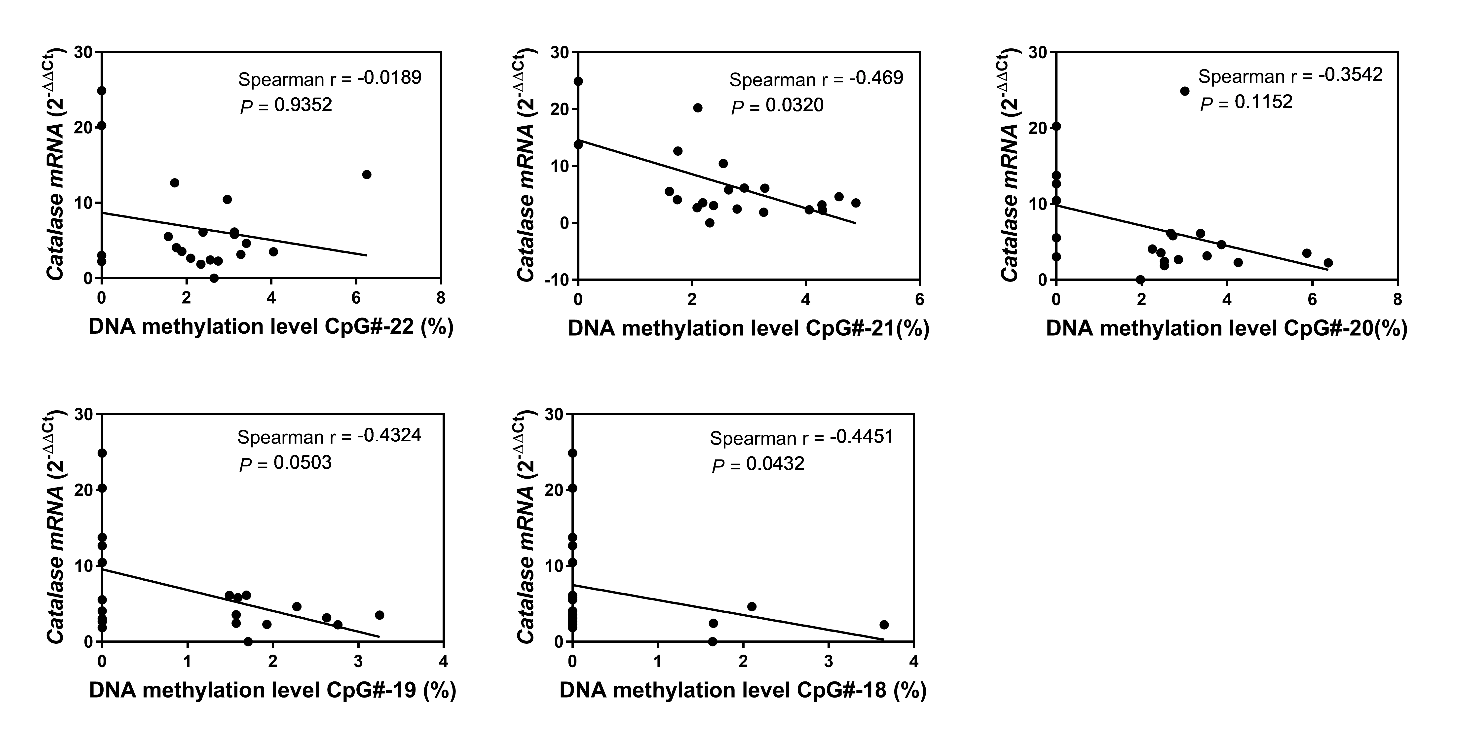
**Fig. 6**

**Fig. 6** Association of DNA methylation percentage of single sites, from CpG-#22 to CpG-#18, with *CAT* mRNA expression in CLL (n = 21).

**
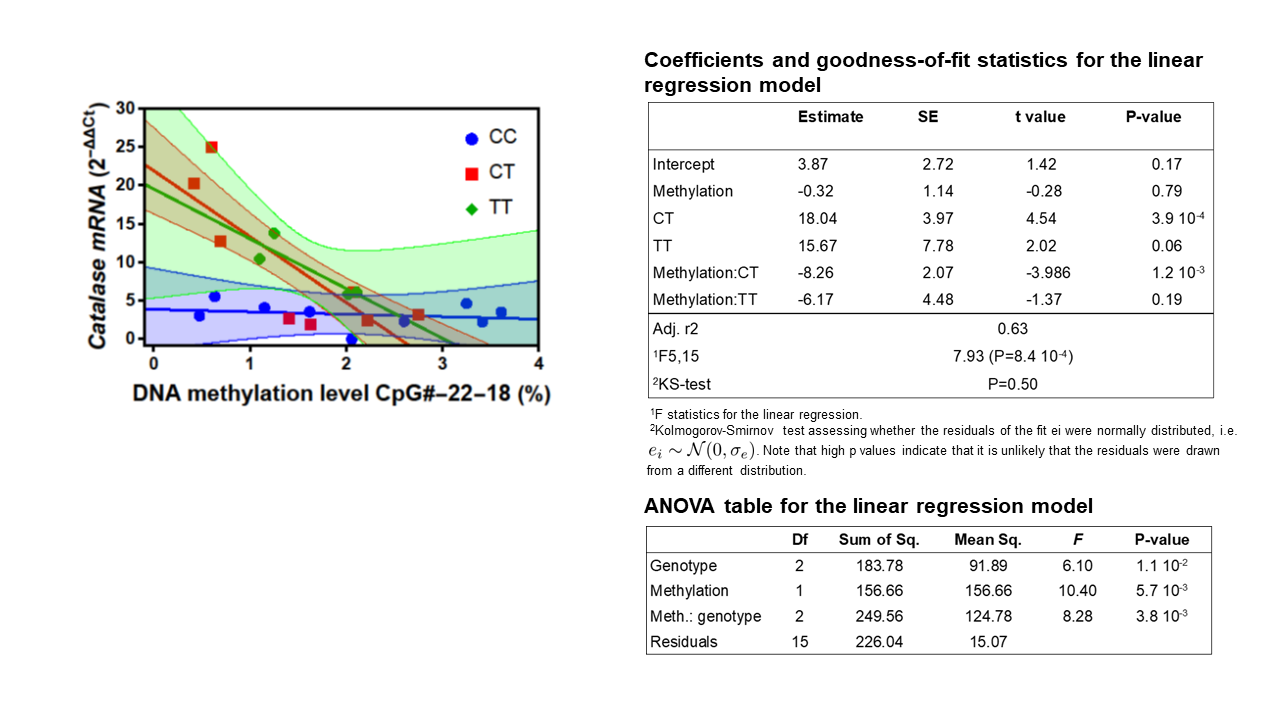
Fig. 7**

**Fig. 7** Interaction between *CAT* promoter genotypes and methylation levels on catalase mRNA expression. The interaction has been investigated within the context of linear models. The figure shows the regression results when methylation on islands CpG#22 to CpG#18 was averaged and stratified for the three genotypes CC, CT, and TT. The line shows the marginal effects (i.e., predicted values) for the significant interaction between genotypes and methylation on mRNA expression. Shaded colored areas indicate the 95% confidence intervals for all interactions. Measurements stratified by genotypes are also shown (points).
